# Body shape matters: Evidence from machine learning on body shape-income relationship

**DOI:** 10.1371/journal.pone.0254785

**Published:** 2021-07-30

**Authors:** Suyong Song, Stephen Baek

**Affiliations:** 1 Department of Economics & Finance, University of Iowa, Iowa City, Iowa, United States of America; 2 Department of Industrial and Systems Engineering, University of Iowa, Iowa City, Iowa, United States of America; University of Luxembourg and Luxembourg Institute of Socio-Economic Research (LISER), LUXEMBOURG

## Abstract

The association between physical appearance and income has been of central interest in social science. However, most previous studies often measured physical appearance using classical proxies from subjective opinions based on surveys. In this study, we use novel data, called CAESAR, which contains three-dimensional (3D) whole-body scans to mitigate possible reporting and measurement errors. We demonstrate the existence of significant nonclassical reporting errors in the reported heights and weights by comparing them with measured counterparts, and show that these discrete measurements are too sparse to provide a complete description of the body shape. Instead, we use a graphical autoencoder to obtain intrinsic features, consisting of human body shapes directly from 3D scans and estimate the relationship between body shapes and family income. We also take into account a possible issue of endogenous body shapes using proxy variables and control functions. The estimation results reveal a statistically significant relationship between physical appearance and family income and that these associations differ across genders. This supports the hypothesis on the physical attractiveness premium in labor market outcomes and its heterogeneity across genders.

## Introduction

In studies on the association between physical attractiveness and labor market outcomes, height, weight, and body mass index (BMI) have been popular choices, as measurements of physical appearance. For instance, Persico *et al*. [1] and Case and Paxson [2] analyzed the association between height and wages. They found apparent height premium in the labor market outcomes. Cawley [3] estimated the effects of BMI on wages and reported that weight lowers the wages of white females. Hamermesh and Biddle studied the impact of facial attractiveness on wages and demonstrated significant beauty premium [4]. However, these measurements of physical appearance are acquired through subjective survey responses. This presents a possibility of attenuation bias from reporting errors on physical appearance, in estimating the relationship between physical appearance and labor market outcomes. In addition, measurements such as height, weight, and BMI are too sparse to characterize detailed body shapes (see Kan and Lee [5]). Consequently, measurement errors in the body shapes would make it difficult to correctly estimate the true relation.

We investigate the properties of reporting errors using a nonparametric conditional mean and nonlinear quantile functions. The nonparametric estimation of the conditional expectations of the reporting errors in height, given the true height, shows that the reporting error for female height is nonclassical in that the reporting error and the true height are dependent. The quantile regression provides that the conditional median of the reporting error is independent of the true height. Thus, it would be more plausible to impose a restriction on the conditional quantile of the reporting error of height than the conditional mean (see Bollinger [6]; Hu and Schennach [7]; and Song [8]). In contrast, the nonparametric conditional mean and nonlinear quantile regressions show that there are substantial nonclassical errors in the reported weight of both genders.

The estimation results for the association between height (or BMI) and family income confirm that the reporting errors have substantial impacts on the estimated coefficients. Furthermore, such classical measurements of body shape are too sparse to describe the whole-body structure. Therefore, analyses with sparse measurements are very sensitive to variable selection, implying that regressions with the measured height and BMI might suffer from measurement errors of body shape. A handful of papers address this problem by proposing statistical methods such as bias-correction methods or instrumental-variables approaches that deliver consistent estimators at the expense of strong assumptions.

The dataset encloses digital 3D whole-body scans of subjects, which is a very unique feature. 3D whole-body scans have been studied in areas such as nutrition/obesity research [9–11], medicine/nursing [12, 13], psychology/cognition of appearance [14, 15], ergonomics/wearable product design [16], and more. However, this study is the first to use 3D whole-body scan data for modeling and analyzing the relationship between family income and body shapes. Indeed, we argue that the 3D scan data of human body shapes would mitigate problem of measurement errors. As the observed variable for body shapes in the dataset is three-dimensional, it is challenging to incorporate the data into the family income model. For this, we adopt methods based on machine learning to identify important features using 3D body scan data. Autoencoders are a certain type of artificial neural network, which possess an hourglass shaped network architecture. They are useful in extracting intrinsic information from high dimensional input and finding the most effective way of compressing such information into the lower dimensional encoding. As this study shows, the graphical autoencoder can effectively extract the features of the body and is not sensitive to random noises.

In economic studies, the focus on non-Euclidean data, such as human body shapes, geographical models, social network data, and so on, is increasing. In this study, we introduce a new methodology developed on deep neural networks and demonstrate its use in analyzing the economic model when the available data has a non-Euclidean structure. The challenge in incorporating non-Euclidean data in statistical analyses is that the data has no trivial grid-like representation. Consequently, encoding the features and characteristics of each data point into a numerical form is neither straightforward nor consistent. Most existing studies simplify the non-Euclidean features with some sparse characteristics. For instance, many relevant studies quantify the geometric characteristics of a human body shape with some sparse measurements, such as height and weight. However, such methods do not always capture detailed geometric variations, and often lead to an incorrect statistical conclusion because of the measurement errors. As a better alternative, we propose a graphical autoencoder that can interface with the three-dimensional graphical data. The graphical autoencoder permits the incorporation of non-Euclidean manifold data into the economic analyses. As we will discuss, the direct incorporation of the graphical data can reduce measurement errors, because graphical data, in general, provides more comprehensive information on non-Euclidean data than do discrete geometric measurements.

Using the graphical autoencoder, we successfully identify intrinsic features of the body shape using 3D body scan data. Interestingly, the intrinsic features of the body type are significant in explaining family income. Using the graphical autoencoder, we identify two intrinsic features forming the male body type and three intrinsic features for the female body type. For both genders, we show that the first feature captures stature, while the second feature captures obesity. The third feature captures the hip-to-waist ratio of the body shape in the female sample. In contrast to the conventional principal component analysis (PCA), the graphical autoencoder allows us to interpret the extracted features. Furthermore, PCA is a linear transformation of the coordinates in the data space, while the autoencoder captures a nonlinear embedding of the data distribution. As Baek [17] and Freifeld *et al*. [18] note, the space in the 3D human body scans exhibits properties of nonlinear manifolds. Hence, the intrinsic features captured by the graphical autoencoder should be a more accurate parameterization of the true data distribution.

As acknowledged in the literature, body types could be endogenous, in that they can be driven by unobserved factors of income such as nutrition, personality, ability, and family background. To identify the relation between body types and family income, we correct for possible endogeneity issues among body types. Dealing with both endogeneity and measurement errors simultaneously in the nonlinear models is not as straightforward as in the linear models (see Song [8]; Song *et al*. [19]; Kim *et al*. [20]; and Kim and Song [21] for detailed discussions). We utilize the proxy variables approach and control functions approach. In particular, our identification strategy is to use variations in shoe size, jacket size for males (blouse size for females), and pants size, as legitimate instrumental variables for stature in the control functions approach. Testing the null of exogenous stature, we find that the female’s stature is endogenous but not the male’s.

**Fig 1:**
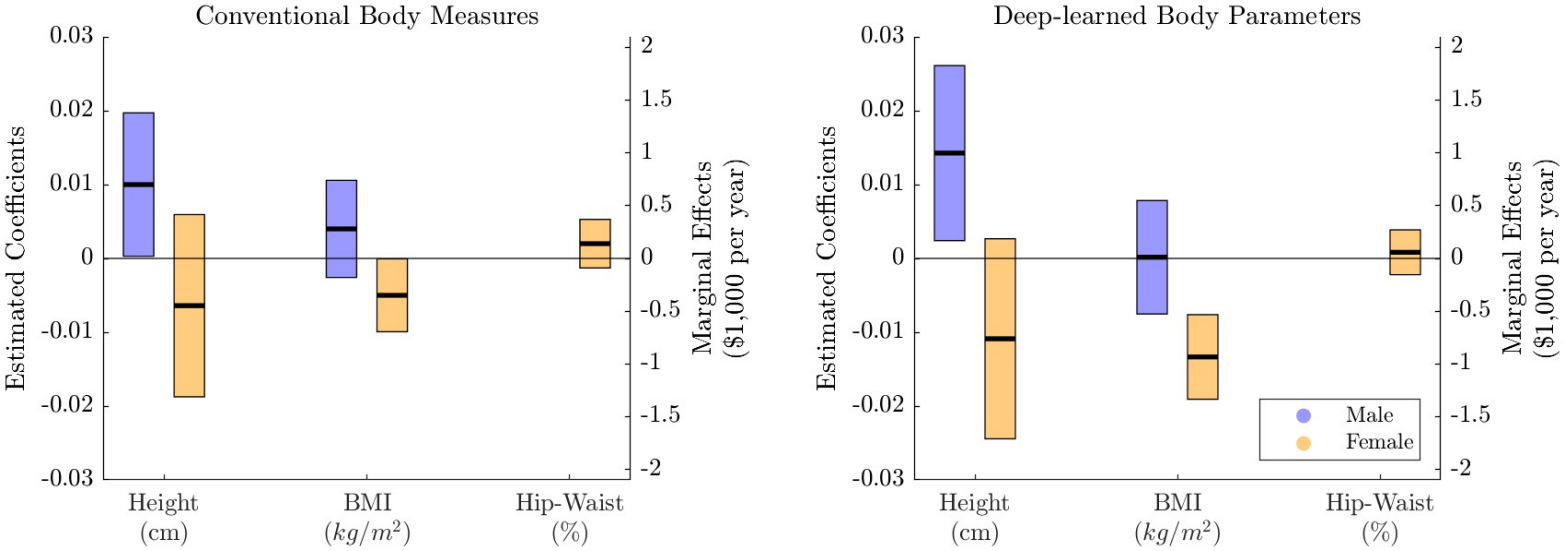
Summary of the estimation results for family income equation. Estimated coefficients and bootstrapped 90% confidence bands are reported. The left panel presents results from the conventional body measures and the right panel reports results from the deep-learned body parameters through the graphical autoencoder.

We summarize the main findings in Fig 1. In the estimation results from the deep-learned body parameters (right panel), we find that the male’s stature has a positive impact on family income and is statistically significant at the 5% significance level, while obesity is insignificant. We estimate that one centimeter increase in stature (converted in height) is associated with approximately $998 increase in family income for a male who earns $70, 000 of the median family income. For females, the coefficient of obesity is negative and statistically significant at the 1% significance level. In contrast, the coefficients of other features such as height and hip-to-waist ratio are statistically insignificant. One unit decrease in obesity (converted in BMI) is associated with approximately $934 increase in the family income for a female who earns $70, 000 of family income. The results show that physical attractiveness premium continues to exist, and the relationship between body shapes and income is heterogeneous across genders, even after controlling for unobserved confounding factors. Education is statistically significant for both genders; however, experience is significant only for the female samples.

Interestingly, the corresponding estimation results from classical body measures such as height, BMI, and hip-to-waist ratio (left panel) are substantially different than those from deep-learned body parameters. For males, the coefficient of height is positive and statistically significant at the 10% significance level: one centimeter increase in height is associated with $700 increase in the family income for a male who earns $70, 000 of the median family income, which is smaller than the effect from the deep-learned body parameters. For females, coefficients of height, BMI, and hip-to-waist ratio are all statistically insignificant. Thus, we do not find strong evidence of body shape effects when using the classical body measures among females. As discussed in the Supporting information, the estimation results with the classical body measures are volatile across different regression models (i.e., OLS, proxy variable approach, and control functions approach), while those with the deep-learned body parameters are robust across different models. These suggest that the classical body measures have limited powers to describe nonlinear body shapes; therefore, any statistical analysis based on those simple measurements would lead to wrong economic inferences. Our findings also highlight the importance of correctly measuring body shapes to provide adequate public policies for improving healthcare and mitigating discrimination and bias in the labor market.

## Model and data

We consider the association between family income and body shapes as follows:

$$\begin{array}{c} \text{Family}\;{\text{Income}}_{i}=\alpha X_{i}+\beta \text{Body}\;{\text{Shapes}}_{i}+\epsilon_{i},\;i=1,\ldots ,N, \end{array}$$ (1)

where Family Income_*i*_ is log family income, Body Shapes_*i*_ is a measure of body types, and *ϵ*_*i*_ is the unobserved causes of family income for individual *i*, and where *X*_*i*_ is a set of covariates, including experience, squared experience, race, occupation, education, marital status, and number of children. We are particularly interested in the parameter *β*, but we also discuss the relationship between family income and other individual characteristics through the vector of parameters *α*. For conciseness, we only report part of the estimated parameters in the tables and complete tables are available upon request. Most of the literature on beauty, height, and weight focuses on the relationship between these factors and individual income or earnings. Because the data used in this study do not contain individual income, we primarily examine the relationship between body shapes and family income. This opens up additional channels through which physical appearance could affect family income. We identify the combined association between body shapes and family income through the labor market and marriage market. In fact, as documented in Chiappori *et al*. [22], various studies found assortative matching on income, wages, education, and anthropometric characteristics such as weight or height in the marriage market. Consequently, the total effects of body shapes in the labor market and marriage market are identifiable from family income, therefore, it is important to investigate the relationship between physical attractiveness and family income.

A large body of literature has analyzed the presence of earnings differentials based on physical appearance. A strand of literature has focused on facial attractiveness. Hamermesh and Biddle analyzed the association between physical appearance and earnings using interviewers’ ratings of respondents’ physical appearance [4]. They found evidence of a positive relationship between looks and earnings. Mobius and Rosenblat examined the sources of beauty premium and decomposed the beauty premium that arises during the wage negotiation process between employer and employee in an experimental labor market [23]. They identified transmission channels through which physical attractiveness raises an employer’s estimate of a worker’s ability. Scholz and Sicinski studied the association between facial attractiveness and lifetime earnings and found that the beauty premium exists even after controlling for other factors that enhance productivity in the labor market earnings [24].

Other threads of literature have analyzed the effects of height on labor market outcomes. Persico et al. [1] found that an additional inch of height is associated with an increase in wages, which is a consistent finding in the literature, in addition to racial and gender bias. They showed that a person’s height as a teenager is the source of the height wage premium. This implies that there are positive effects of social factors associated with the accumulation of productive skills and attributes on the development of human capital and the distribution of economic outcomes. Case and Paxson also found substantial returns to height in the labor market [2]. However, they showed that the height premium is the result of positive correlation between height and cognitive ability. Lundborg et al. found that the positive height-earnings association is explained by both cognitive and noncognitive skills observed in tall people [25]. Deaton and Arora reported that taller people evaluate their lives more favorably and their findings are explained by the positive association between height and both family income and education [26]. Böckerman and Vainiomäki used twin data to control for unobserved ability and found a significant height premium in wage for women but not for men [27]. Lindqvist studied the relationship between height and leadership and confirmed that tall men are significantly more likely to attain managerial positions [28].

Cawley considered the effects of obesity on wages [3]. He found that weight lowers the wages of white females and noted that one possible reason for this is that obesity has adverse impact on the self-esteem of white females. In a similar model, Kan and Lee showed that BMI is not an appropriate measure of obesity and proposed flexible econometric approaches allowing for nonlinear relation [5]. Rooth used a field experimental approach to find differential treatment against obese applicants in terms of the number of callbacks for a job interview in the hiring process in the Swedish labor market [29].

Many relevant studies in the literature on physical appearance quantify the geometric characteristics of a human body shape with some sparse measurements, such as height, weight, or BMI. However, as we will see in later sections, such quantification methods do not always capture detailed geometric variations, and often lead to an erroneous explanation of statistical data. For instance, with height and BMI alone, one can hardly distinguish muscular individuals from individuals with round body shapes. The situation does not improve even if some new variables, such as chest circumference, are added, as these variables are still insufficient to codify all the subtle variations in body shapes. Moreover, often, such additional variables merely add redundancy, without adding any substantial statistical description of data, as the commonly-used anthropometric parameters are highly correlated to each other. In addition, notably, the manual selection of measurement variables can also introduce one’s bias into the model. In this study, we compare several common ways of quantifying manifold structured data with a newly-proposed graphical autoencoder method.

We use a unique data, called the Civilian American European Surface Anthropometry Resource (CAESAR) dataset. It was collected from a survey of the civilian populations of three countries representing the North Atlantic Treaty Organization (NATO) countries: the U.S., the Netherlands, and Italy. The survey was primarily conducted by the U.S. Air Force and we used the sample from the U.S. for our study. The survey for the U.S. sample was conducted from 1998 to 2000 and carried out in 12 different locations, which were selected to obtain subjects approximately in proportion to the population in each of the four regions of the U.S. Census. Survey sites include LA (CA), Detroit (MI), Ames (IA), Dayton (OH), Greensboro (NC), Marlton (NJ), Ottawa (Ontario, CAN), Minneapolis (MN), Houston (TX), Portland (OR), San Francisco (CA), and Atlanta (GA). The U.S. data is referred to as the North American sample, as one site in Ottawa, Canada, was added to the sample.

The dataset contains 2,383 individuals, whose ages vary from 18 to 65, with a diverse demographical population. The dataset contains detailed demographics of subjects, anthropometric measurements done with a tape measure and caliper, and digital 3D whole-body scans of subjects. In contrast to other traditional surveys, the data contains both reported and measured height and weight. This feature makes it possible to calculate reporting errors in the survey data and analyze their relations to the correctly measured height/weight as well as individual characteristics. In addition, the existence of 3D whole-body scan data enables the CAESAR data to serve as a good proxy to physical appearance, such that the potential issue of measurement errors can be mitigated.

## Reporting errors in height and weight

Several studies in the literature use survey data, and therefore, they assume there are no reporting errors in height and weight, or that reporting errors are classical in that they are not correlated with true measures. Exceptionally, Persico et al. [1] and Case and Paxson [2] use measured height from the British National Child Development Survey, although they also use self-reported height from the British Cohort Study and the National Longitudinal Survey of Youth, respectively. Lundborg et al. [25] use measured height from the Swedish National Service Administration. As our data contains both reported and measured height and weight, we can further investigate the properties of the reporting errors. We consider measured height and weight as the true height and weight, as they are measured by professional tailors. The reporting errors are calculated as Reporting Error^*H*^ = Reported Height − Height and Reporting Error^*W*^ = Reported Weight − Weight, respectively.

**Fig 2:**
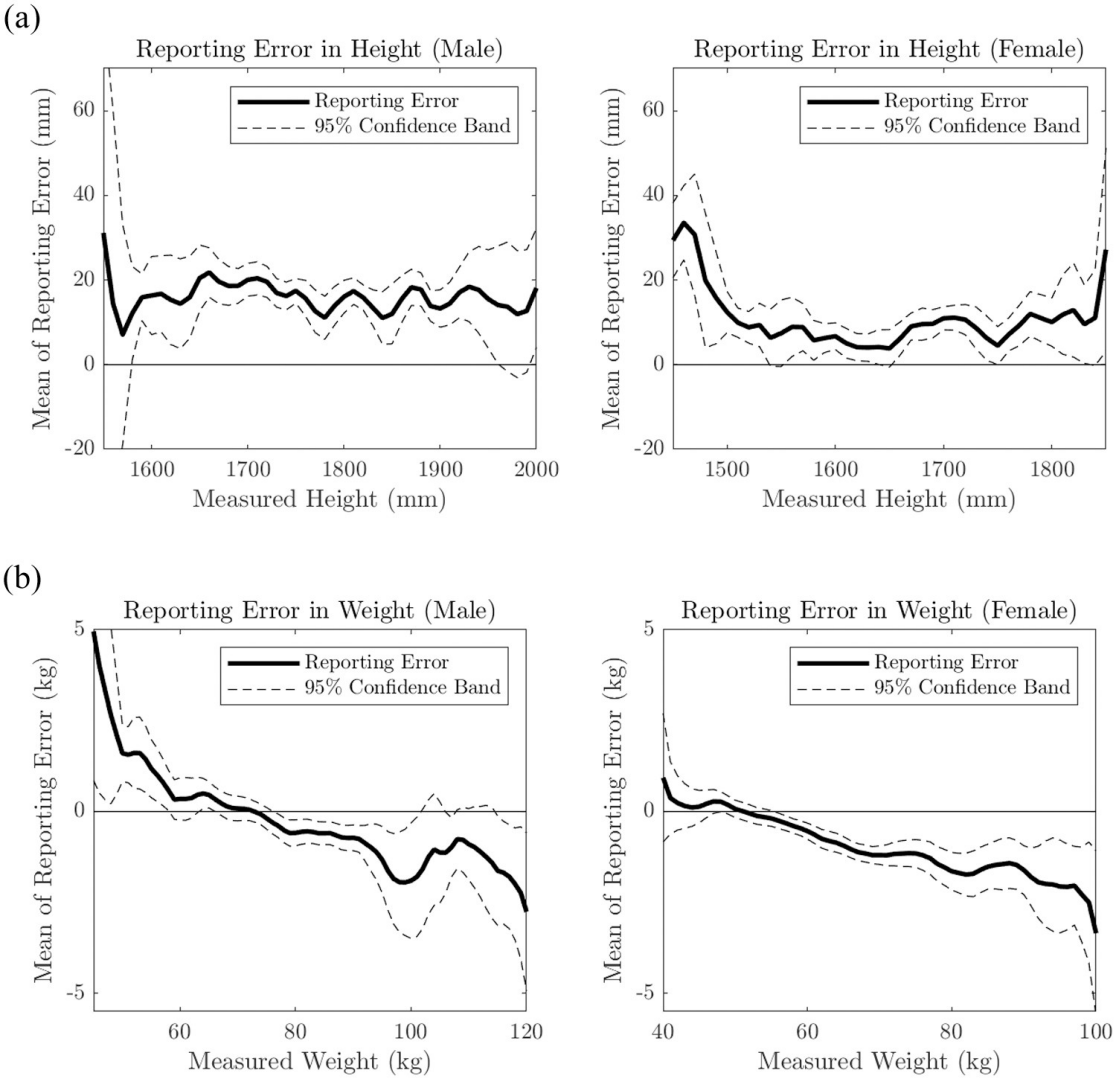
Relationship between reporting error and true measure. Conditional mean of reporting error in height conditional on true height (top) and reporting error in weight conditional on true weight (bottom).

We test whether the reporting errors depend on true values of height or weight. Fig 2 plots the estimation of the conditional expectations of the reporting errors in height/weight given the true measures, namely, *E*[Reporting Error^*H*^ ∣ Height] and *E*[Reporting Error^*W*^ ∣ Weight] with their 95% confidence bands, respectively. We use nonparametric kernel estimation, where the kernel function is an Epanechnikov kernel and the bandwidth is chosen using Silverman’s rule-of-thumb. The confidence bands are estimated by a nonparametric bootstrap method. The solid line represents zero reporting error. The nonparametric plots for height show that reported height is larger than true height at almost all height levels in both genders, showing over-reporting patterns. It displays no significant relation between the reporting error and the true height for males. For females, we observe more reporting error at low height levels than at the average height level, which indicates that the reporting error for female height is nonclassical in that the reporting error and the true height are dependent. This result is a finding that is not captured by the linear mean regression, where the reporting error in height is not related to the true height for both genders (see the Supporting information for linear mean regression and quantile regression).

The plots for the reported weight show more substantial nonclassical errors. Males at low weight levels (below approximately 75 kilograms) have a tendency to over-report their weight; however, males above approximately 75 kilograms under-report their weight. Similarly, females at the low weight levels (below approximately 50 kilograms) have a tendency to over-report their weight; however, females at weight levels above approximately 50 kilograms under-report their weight. Both plots display an apparent dependence between the reporting error and the true weight. This confirms the significant negative relation between the reporting error and the true weight shown in the linear mean regression provided in the Supporting information.

## Estimation of the association between physical appearance and labor market outcomes

In this section, we estimate the association between the physical appearance and family income using various methods. As expected, the reporting errors and measurement errors in body shapes have significant impacts on the estimated outcomes. We propose a machine learning method to control for them.

### Height, weight, and reporting errors

As in Cawley [3], we consider BMI as a proxy to the body shapes in the regression Eq (1) to estimate the association between obesity and income. We add height or weight as an additional regressor to take into account a possible omitted variable problem. Therefore, we consider the income equations as follows:

$$\text{Family}\;{\text{Income}}_{i}=\alpha X_{i}+\beta_{1}{\text{BMI}}_{i}+\epsilon_{i},$$ (2)

$$\text{Family}\;{\text{Income}}_{i}=\alpha X_{i}+\beta_{1}{\text{BMI}}_{i}+\beta_{2}{\text{Weight}}_{i}+\epsilon_{i},$$ (3)

$$\text{Family}\;{\text{Income}}_{i}=\alpha X_{i}+\beta_{1}{\text{BMI}}_{i}+\beta_{2}{\text{Height}}_{i}+\epsilon_{i},$$ (4)

where BMI_*i*_ is the body mass index. We note that family income could suffer from reporting error in the survey data. Nevertheless, the reporting error in the dependent variable only increases the variance of the estimator, so long as the reporting error is uncorrelated with the regressors, which in our opinion is a reasonable assumption. Thus, we primarily focus on the reporting error in the proxies to the body shapes. We first estimate the equations using the reported variables and summarize the estimation results in the Supporting information. The columns for males in S1 Table show that the coefficient of the reported BMI in Eq (2) is statistically insignificant. Adding the reported weight or height does not change the result for the reported BMI, as in Eqs (3) and (4). Instead, the estimated coefficient of the reported height or weight is significant. For females, the coefficients of the reported BMI are insignificant in Eqs (2) and (4). However, in Eq (3), the reported BMI is negatively correlated with family income, and the relation is statistically significant at the 1% significance level. It also shows that the coefficient of the reported height or weight is positive and significant at the 5% significance level.

**Fig 3:**
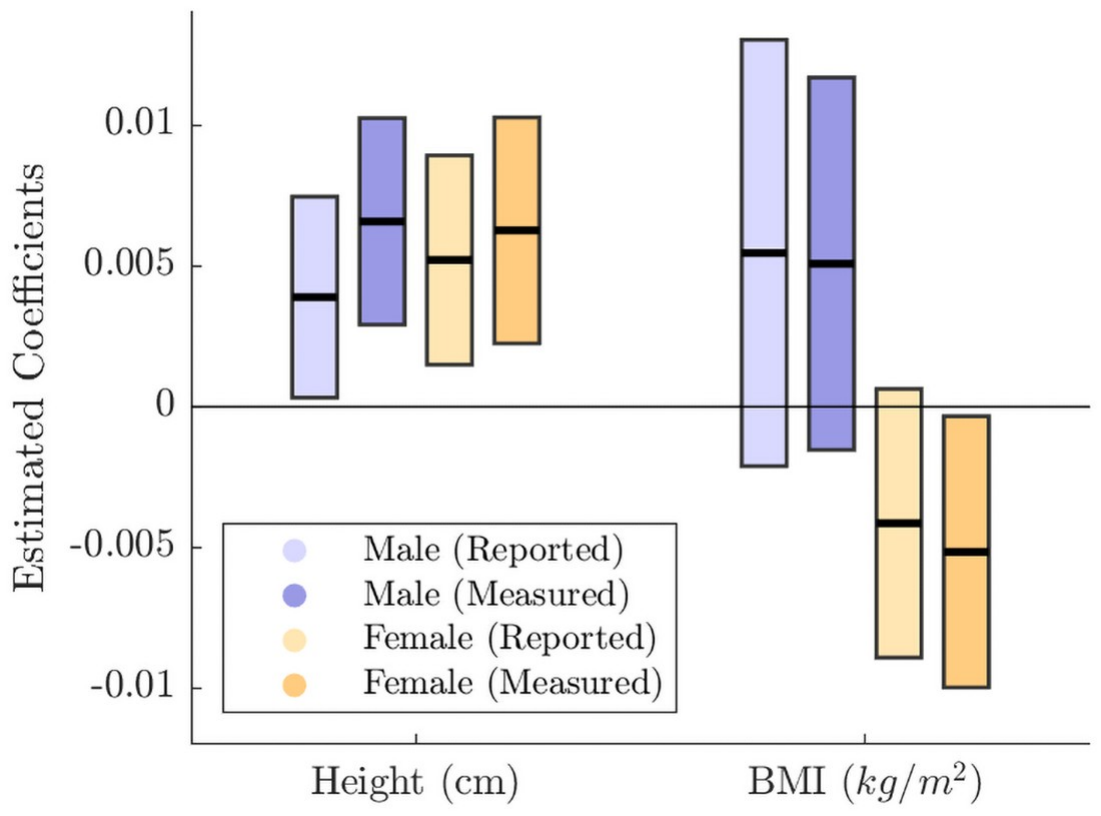
Comparison of reported and measured body measures. We report estimated coefficients and bootstrapped 90% confidence bands. We provide results from reported body measures (Reported) and measured body measures (Measured). Note that the unit for height is converted into centimeter (cm).

We next estimate Eqs (2)–(4) using the measured BMI, height and weight. As shown in S2 Table, the estimation results are somewhat different from those with the reported variables for all equations. In Eq (2) for males, the coefficient of BMI is still insignificant. When weight is included, as in Eq (3), its coefficient for males is positive and statistically significant at the 1% significance level. The coefficient of BMI becomes negative and statistically significant at the 5% significance level. When height is included, as in Eq (4), its coefficient for males is positive and statistically significant at the 1% significance level. However, the coefficient of BMI is statistically insignificant. For females, the results are also different from those in the regression with the reported variables. The coefficient of BMI is always negative and statistically significant. The coefficient of height or weight is positive and statistically significant. The results for Eq (4) are highlighted in Fig 3. It shows that the magnitudes of the coefficients for height are larger when using measured height. For females, the impact of the measured BMI on family income is significant in contrast to the insignificant impact of the reported BMI. Different signs on the effect of BMI across genders are also observed: the positive effect of male BMI as opposed to negative effect of female BMI. Thus, the analysis confirms that the reporting errors in body measures have significant impacts on the estimated coefficients. In the Supporting information, we provide a similar analysis on the regression of family income on height and weight.

Interestingly, we also observe that the estimation results have significantly changed, as different sets of measures of body types were included in the equations. One possible explanation for this is that even the measured height and BMI might not be perfect proxies to the body types, although they are less prone to reporting errors. In fact, height, weight and BMI are simple measures of body types, and they might miss useful information on true body types (e.g., see Kan and Lee [5] and Wada and Tekin [30] for BMI). Several studies propose statistical methods to reduce the measurement errors in body-shape measurements. Among others, Hu and Sasaki [31] propose closed-form estimators for nonparametric regressions of obesity on health care usage, using clinical measurements and self-reporting of BMI. Courtemanche et al. [32] propose a rank-based correction method for using validation data to correct the measurement errors in obesity. Murillo et al. [33] reduce bias in obesity by applying regression calibration, simulation extrapolation, and multiple imputation approaches. We further investigate the role of the measurement errors using 40 body measurements in place of BMI and height, and the results confirmed that BMI, height, and weight cannot fully describe body shapes (see the Supporting information for more details).

### Physical appearance and graphical autoencoder

The characterization of geometric quantities, such as the physical appearance of human body shapes using a sparse set of canonical features (e.g., height and weight) often causes unwanted bias and misinterpretation of data. For simple shapes such as rectangles, canonical measures such as width and height, already provide a complete description of the shape. Hence, shape variation among rectangles could easily be described using the two canonical parameters without much issues. However, this seldom applies to more sophisticated shape variations, if at all. Instead, the canonical shape descriptors, often hand-selected, might cause *nonignorable* error in capturing genuine statistical distribution by overlooking some important geometric features or measuring highly-correlated variables redundantly, which can be considered as a measurement error of some sort.

Unfortunately, however, extracting a complete and unbiased set of shape descriptors is not a trivial task. Furthermore, the task is highly problem-specific, such that, for example, the shape descriptors for car shapes would not be appropriate for describing human body shapes. Therefore, in this study, we propose a novel data-driven framework for extracting complete and unbiased shape descriptors from a set of geometric data. The proposed framework utilizes an autoencoder neural network (Bourlard and Kamp [34]) defined on a graphical model. We demonstrate that the shape descriptors obtained through the new approach can actually provide a better description of data.

**Fig 4:**
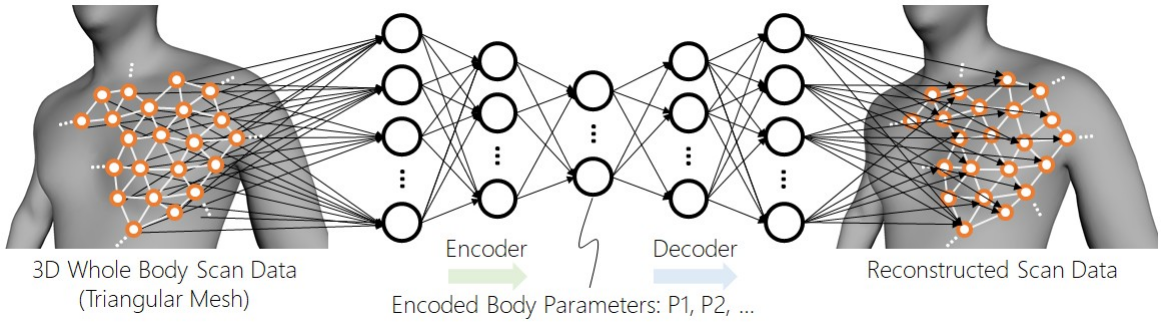
Schematic illustration of the proposed graph autoencoder. A discrete-sampled scalar field acts as input and output nodes of the autoencoder. The intermediate layers are similar to the ordinary autoencoder layers.

Fig 4 illustrates a schematic overview of the graphical autoencoder. As the figure shows, the vertices of a topology-normalized graphical model act as input neurons in the autoencoder model. Then the input neurons are connected to the hidden neurons in the next layer, which then are connected in chain through the “bottleneck” layer. The bottleneck layer has a significantly smaller number of neurons than the input neurons; hence, the dimensionality compression occurs there. The latter half of the autoencoder is symmetric to the first half and finally reconstructs the bottleneck encoding into the original graphical model. The training process of the graphical autoencoder attempts to minimize the discrepancy between the reconstructed model and the original input by tuning the neural weights of the hidden layers.

#### Graphical autoencoder on CAESAR dataset

The CAESAR scan dataset includes 15, 178 vertices as well as (*x*, *y*, *z*) coordinate. This gives us 45, 534 inputs for each individual. To extract body shape parameters that encode the geometric characteristics of a person’s appearance, we designed a graphical autoencoder consisting of seven hidden layers. Each hidden layer comprises 256–64-16-*d*-16–64-256 neurons, respectively, where *d* is the intrinsic data dimension, or the dimensionality of the embedding. We used the RMSprop optimizer for training. The dataset was randomly split to a training group used for training and a validation group that was set aside during the training. The ratio between the number of data samples in such groups were 80:20, respectively. The training continued until 5,000 epochs with a batch size of 200 samples. As a criterion to evaluate the performance of the graphical autoencoder, we used the reconstruction error measured in mean-squared-error (MSE). As described above, the graphical autoencoder first embeds graphical data into a lower dimensional embedding through the encoder part of the network, which is then reconstructed into a graphical model through the decoder part. We compared the differences in the reconstructed output and the original input to the network.

When using the full sample, we found three intrinsic features of body shapes. In particular, the third feature was associated with femininity/masculinity. Based on such observation, we conducted another similar experiment for training the graphical autoencoder with separate genders. Among 2,383 subjects in the CAESAR dataset, there were 1,122 males and 1,261 females. The two groups had been separated into two experiment sessions, in which they were further separated into training and validation groups with the same 80:20 ratio. The experiment with separate genders demonstrated a similar trend as the experiment with a full sample in terms of the effect of the intrinsic dimension on the reconstruction error, as visualized in S1 Fig. However, interestingly, this time, the reasonable dimension *d* of intrinsic parameters was observed to be 2 for male subjects against 3 in the full sample case. We interpret this result as follows: as the two genders are now separated, the role of the third parameter (femininity/masculinity) is now less significant than before in the full sample case, and thus, the gain in accuracy by including the third parameter in addition to the two parameters becomes negligible for males. However, such interpretation was not true for the female population, as the accuracy was in fact higher when the third parameter was included. This is because female body shapes have greater variation in body curves than males, and therefore, the third component has greater significance for females. Therefore, we select *d* = 2 for males and *d* = 3 for females. Lastly, we also note that the convergence was slower when the two genders were separated and a measurable gain in accuracy could be observed even after 1, 000 epochs, which was not the case when the two genders were combined in training. This could be because the number of training samples in the training dataset is significantly smaller (about a half) than the previous case, rendering a drop in the representative power of the data.

S2 Fig illustrates the body shape spanned by the two parameters obtained from the graphical autoencoder for each gender. 3D body shape models are arranged in accordance with their body shape parameters with increments of −3*σ*, −1.5*σ*, 1.5*σ*, and 3*σ* with respect to the mean in each direction, where *σ* is the standard deviation of each parameter. Body shapes for male (left) and female (right) display similar patterns over changes in the two parameters. Overall, the first parameter *P*_1_ affects the height of a person. That is, a smaller value in *P*_1_ indicates the person is not tall compared to the other population and vice versa. *P*_2_ is how lean a person is. That is, a large value in *P*_2_ results in an obese person, while a small value in *P*_2_ results in a more slim and fit person.

To better understand these parameters, we consider a linear fit of BMI, height, or weight on each parameter. S3 Fig shows the relationship between body shape parameters and the classical body measurements for males. *P*_1_ is positively correlated with BMI, height, and weight. Among these body measurements, height is the most highly correlated with *P*_1_ (approximately *R*^2^ = 0.70). *P*_2_ is negatively correlated with height, but is positively correlated with BMI and weight. BMI has the highest correlation with *P*_2_ (approximately *R*^2^ = 0.69). S4 Fig displays the relationship between body shape parameters and the classical body measurements for females. The patterns are close to those for males in S3 Fig. As discussed, the female sample produces an additional feature, *P*_3_. We visualize the third parameter for females in S5 Fig. As the figure shows, *P*_3_ captures the ratio of hip to waist for females, which is unique to the female dataset. For simplicity, we will interpret *P*_1_, *P*_2_, and *P*_3_, as features associated with a person’s stature, obesity, and hip-to-waist ratio, respectively.

**Fig 5:**
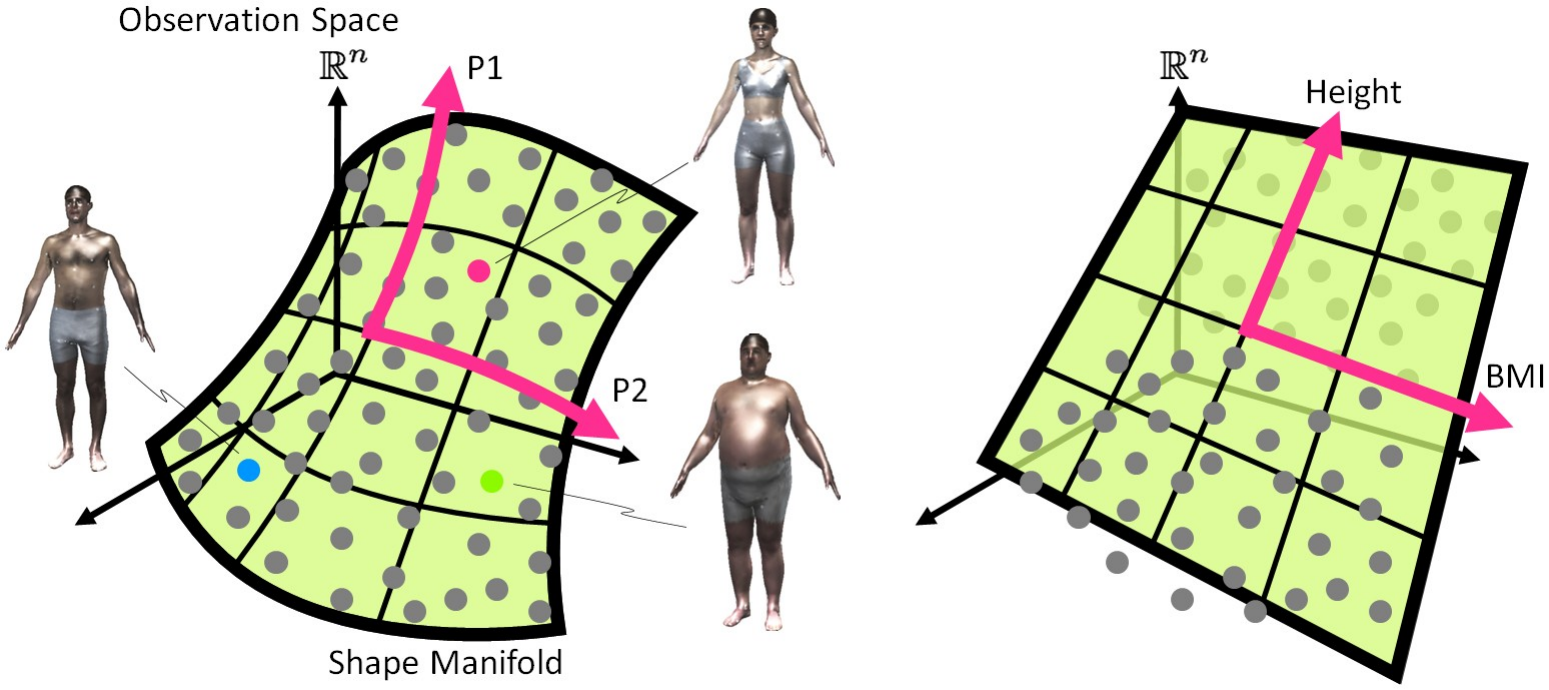
Graphical illustration of (*P*_1_, *P*_2_) and the classical proxies.

Note that the extracted features *P*_1_, *P*_2_, and *P*_3_ perform better in explaining nonlinear variations in body shapes than simple measures such as height, BMI, and hip-to-waist ratio. In contrast to the classical proxies, *P*_1_, *P*_2_, and *P*_3_ nonlinearly encode the space of 3D body shapes (Fig 5). From evidence provided by Baek [17] and Freifeld *et al*. [18], we argue that such a nonlinear parameterization better approximates the actual data manifold. As shown in S2 Fig, the body shape spanned by *P*_1_ and *P*_2_ displays dynamic and nonlinear patterns as the parameters vary. In addition, we consider a linear prediction of female *P*_3_ using 40 various body measures provided in S3 Table and report statistically significant variables in S6 Fig. It shows that many body parts are highly associated with *P*_3_. This confirms that *P*_3_ captures complexity in female body shapes and reflects not only hip-to-waist ratio but also variations in other body parts. Nevertheless, we call *P*_3_ hip-to-waist ratio for convenience.

A reviewer suggested that the various body measures could be used as input neurons in place of 3D scanned data in the autoencoder model. In fact, the extracted features from the various body measures were better than three classical proxies in describing body shapes and explaining family income. Nevertheless, they had limited interpretability, as they were unable to display graphical rendering, as in S2 and S5 Figs. They also failed to capture *P*_3_, hip-to-waist ratio. Moreover, their estimated effects on family income were smaller than those of *P*_1_, *P*_2_, and *P*_3_.

#### Extracted body types and family income

We now use the measurements of body type that are extracted by the graphical autoencoder in the previous section. We estimate the Eq (1) with the extracted body types in place of a set of body measurements for Body_*i*_ as follows:

$$\begin{array}{cc} & \text{Family}\;{\text{Income}}_{i}=\alpha X_{i}+P_{1i}+\epsilon_{i}, \end{array}$$ (5)

$$\begin{array}{cc} & \text{Family}\;{\text{Income}}_{i}=\alpha X_{i}+P_{2i}+\epsilon_{i}, \end{array}$$ (6)

$$\begin{array}{cc} & \left\{ \begin{array}{cc} \text{Family}\;{\text{Income}}_{i}=\alpha X_{i}+\beta_{1}P_{1i}+\beta_{2}P_{2i}+\epsilon_{i} & \text{if}\;\text{male},\\ \text{Family}\;{\text{Income}}_{i}=\alpha X_{i}+\beta_{1}P_{1i}+\beta_{2}P_{2i}+\beta_{3}P_{3i}+\epsilon_{i} & \text{if}\;\text{female}, \end{array}\right. \end{array}$$ (7)

where *P*_1*i*_, *P*_2*i*_ and *P*_3*i*_ are body types for each individual *i*. S4 Table reports estimation results across the gender with the same set of controls. In Eq (7), we add all intrinsic features of the body shape to the income equation. For males, only the coefficient of the *P*_1_ measurement is statistically significant and *P*_2_ is not associated with the family income. One standard deviation increase in males’ *P*_1_ is associated with 0.052 increase in log family income. For females, only the coefficient of the *P*_2_ measurement is statistically significant, and *P*_1_ and *P*_3_ are not correlated with the family income. When these insignificant variables are dropped in Eqs (5) and (6), the regression equations obtain higher adjusted R squared. The results show that one standard deviation decrease in females’ *P*_2_ is associated with 0.056 increase in log family income.

For comparison, we replace the extracted body types with height, BMI, and hip-to-waist ratio, and re-estimate the above equations. Hip-to-waist ratio is calculated as (Hip Circumference, Maximum) ÷ (Waist Circumference, Preferred) × 100. As shown in S5 Table, in both genders, height has positive impact on log family income and is statistically significant. The estimated coefficients of BMI and hip-to-waist ratio are insignificant. In particular, BMI was significant at 10% significance level when height and BMI are included. However, BMI becomes insignificant when hip-to-waist ratio is added. In this case, we observe no gender differential in the impact of body types. The results confirm that the estimation of the income equation with classical body measurements is susceptible to variable-selection and provides different conclusions than those with our proposed method.

### Endogenous body types

If unobserved determinants of family income such as individual ability, personality, and childhood nutrition are correlated with physical appearance, the estimates in the previous section are inconsistent. As well documented in Cawley [35], there is a broad set of causes of obesity; however, most studies are able to correct for only a small portion of the possible factors. We attempt to control for the unobserved determinants of obesity as much as possible using their observed proxies, which are available in the data. Although these unobserved determinants of family income have been controlled for, there would be other possible unobserved factors, such as individual ability, which make individual stature endogenous. As reported in Persico et al. [1], Case and Paxson [2], and Lundborg et al. [25], stature would be highly correlated with individual cognitive and noncognitive abilities that potentially cause family income. We also take into account such possibilities to identify the relationship between body types and family income.

We summarize the estimated coefficients in S6 Table for the classical body measures and in S7 Table for the deep-learned body parameters. They show that the estimated effects from the measured height, BMI, and hip-to-waist ratio are substantially different than those from the deep learned parameters. One can possibly interpret such difference as a limitation of classical body measures on describing appearances. In fact, it is widely known in the literature (CDC document, accessed 2019 [36]) that BMI is a surrogate measure of body fat. Neither does it distinguish fat, muscle, or bone mass, nor does it describe distribution of fat among people. Therefore, it is possible that the difference in family income is falsely correlated with stature, while the true underlying statistics suggests otherwise. To illustrate this problem, consider a tall and visually obese male and a short and muscular male with the same body mass. In this case, as there is no difference in BMI, the difference in family income must be explained by stature, which may lead to an inaccurate conclusion. However, this was not the case for the deep-learned parameters.

## Conclusion

This study examines the relationship between physical appearance and family income. We demonstrate significant reporting errors in the reported height and weight, and show that these discrete measurements are too sparse to provide a complete description of body shape. In fact, we show that these reporting errors are correlated with individual backgrounds. We also find that the regression of family income on the self-reported measurements suffers from reporting errors and delivers biased estimates compared to the regression on the true measurements. The findings reveal the importance of measuring body types instead of simply relying on self-reporting by subjects for public policies.

We introduce a new methodology built on a graphical autoencoder in deep machine learning. Using three dimensional whole-body scan data, we identify two intrinsic features consisting of human body shapes for males and three intrinsic features for females. These body features are presumably less likely to suffer from measurement errors in physical appearances. We also take into account a possible issue of endogenous body shapes by utilizing proxy variables and control functions approaches. The empirical results document the positive impact of stature on family income for males. However, results for females show that obesity is the only significant feature, and it negatively affects family income. The findings support the hypotheses on the physical attractiveness premium and the differential treatment across the gender in the labor market outcomes.

Discrimination based on gender/physical appearance can take place both in the labor market and the marriage market. While the marriage market is beyond the reach of a public policy, as far as the labor market is concerned, the policy implications of this current work should be that (1) efforts to promote the awareness of such discrimination must occur through workplace ethics/non-discrimination training; and (2) mechanisms to minimize the invasion of bias throughout hiring and promotion processes, such as blind interviews, should be encouraged.

The data set was collected in 2002. Society has certainly changed since then, in terms of the perception of beauty, people’s awareness of social biases in the socioeconomic market, regulations and policies, and so on. Therefore, the analysis results may or may not be valid today. Although the scientific contribution of this current work is in the use of a novel dataset and a new methodology, however, the readers should exercise caution in applying the results presented in this paper directly to society today. Instead, to obtain a more accurate view of recent trends, a follow-up study would be necessary with the newest observations and measurements. Note also that marital status is not statistically significant in all regressions for family income throughout this study. Therefore, it is possible that the family incomes reported in the survey data are likely to be individual income. Thus, further investigations with a new survey on individual income would be an interesting direction for the future research.

Finally, we believe that the body scans may also provide some interesting additional details beyond what is presented in this study. For example, geometric measurements of different body parts (e.g. volume of abdomen, curvature of waist line) may lead to a discovery of new trends and correlations with socioeconomic outcomes. We also believe that the proposed method can be applied to many interesting research questions, which deal with geometric data such as graphical, image, spatial, and social networks data. For example, Hoberg and Phillips constructed firm pair-wise similarity scores based on text analysis using product descriptions, to obtain industry classifications [37]. Product similarity was measured by the cosine distance between the word vectors extracted from product descriptions. The idea of graphical autoencoder could be applied, in this case, to encode product descriptions and their similarity relationships so that more precise competitive connections among firms can emerge.

## Supporting information

S1 Appendix Online supplementary appendix. We provide more detailed results and additional analyses to the findings in the main text.
(PDF)

S1 Dataset Reproduceability of the results. We provide the source codes and processed dataset at the following URL: http://bit.ly/shape-matters.
(CSV)

S1 Fig Result of training graphical autoencoder separately on each gender. The abscissa is the number of epochs for the training and the ordinate is the model loss in terms of MSE. The left shows the loss on training dataset (training loss) while the right shows the loss on validation dataset (validation loss).
(TIF)

S2 Fig Body shape parameters derived from the graphical autoencoder. 3D body shape models for male (left) and female (right) are arranged in accordance with their body shape parameters, with increments of -3*σ*, -1.5*σ*, 0, 1.5*σ*, and 3*σ* with respect to the mean in each direction, where *σ* is the s.d. of each parameter.
(TIF)

S3 Fig Relationship between body shape parameters and the classical body measurements for males. The straight line displays the linear fit. The R-squared is reported in the parentheses.
(TIF)

S4 Fig Relationship between body shape parameters and the classical body measurements for females. The straight line displays the linear fit. The R-squared is reported in the parentheses.
(TIF)

S5 Fig The third body shape parameter *P*_3_ for females. The third parameter tends to capture the hip-to-waist ratio of the body shape among the female subsample.
(TIF)

S6 Fig Various measures and *P*_3_ for females. Estimated coefficients and bootstrapped 90% confidence bands are reported for females. Note that units for all measurements, except cup size, are converted into centimeters (cm).
(TIF)

S1 Table The association between reported BMI and family income. (PDF)

S2 Table The association between BMI and family income. (PDF)

S3 Table List of various body measures. (PDF)

S4 Table The association between body-type parameters and family income. (PDF)

S5 Table The association between BMI/height/hip-to-waist-ratio and family income. (PDF)

S6 Table The association between BMI/height/hip-to-waist-ratio and family income—control function approach. (PDF)

S7 Table The association between body-type parameters and family income—control function approach. (PDF)

S1 File (DS_STORE)
