# Supplementary material for: Body shape matters: Evidence from machine learning on body shape-income relationship
Source: PLoS One. 2021 Jul 30;16(7):e0254785. doi: 10.1371/journal.pone.0254785 (PMC8323889; doi:10.1371/journal.pone.0254785)
Supplement: S1 Appendix — We provide more detailed results and additional analyses to the findings in the main text. (PDF) [file pone.0254785.s001.pdf]

Body Shape Matters (Online Supplementary Appendix)

Suyong Song[1*], Stephen Baek[2],

**1** Department of Economics & Finance, University of Iowa, Iowa City, Iowa, United States
**2** Department of Industrial and Systems Engineering, University of Iowa, Iowa City, Iowa, United States

*suyong-song@uiowa.edu

## Abstract

In this supplementary appendix, we provide more detailed results and additional analyses to the findings in the main text.

## Data

The survey of the U.S. sample was conducted from 1998 to 2000 and carried out in 12 different locations that were selected to obtain subjects approximately in proportion to the proportion of the population in each of the four regions of the U.S. Census. The dataset contains detailed demographics of subjects, anthropometric measurements done with a tape measure and caliper, and digital 3D whole-body scans of subjects. Some of the 2,383 subjects in the database have missing demographic and anthropometric information; we have excluded these from our study. In addition, subjects who elected not to disclose and/or were not aware of their income, race, education, were removed from this study. In the analysis, we divide the sample by gender to take into account the differential treatment across genders.

Tables S.1-S.2 provide summary statistics of the variables in the database for males and females, respectively. The data has a single question about family income (grouped into ten classes). Average family income is $76,085 for males and $65,998 for females. The differences in average family income across genders would be because the male sample includes more married people than the female sample. Median family income is slightly lower than the mean family income, which amounts to $70,000 for males and $52,500 for females. For males, on average, reported height is 179.82 centimeters and measured height is 178.26 centimeters, which shows a tendency of over-reporting. The gap is larger when median reported height (180.34 centimeters) and measured height (177.85 centimeters) are compared. We observe a similar pattern in the female sample: reported height is 164.96 centimeters and measured height is 164.22 centimeters on average; median reported height is 165.1 centimeters and median measured height is 164 centimeters.

The males' average reported weight is 86.03 kilograms and the average of the measured weight is 86.76 kilograms. The median of two measurements are the same. For females, reported weight is 67.88 kilograms and measured weight is 68.81 kilograms on average. Median reported weight is 63.49 kilograms and median measured weight is 64.85 kilograms. In both subsamples, the standard errors of the weight are large at approximately 17 kilograms. BMI has been commonly used as a screening tool for determining whether a person is overweight or obese. According to the Centers for

Disease Control and Prevention (CDC), the standard weight status categories associated with BMI ranges for adults are as follows: below 18.5 (underweight), 18.5-24.9 (normal or healthy weight), 25.0-29.9 (overweight), 30.0 and above (obese). BMI is calculated as weight in kilograms divided by height in meters squared. We refer reported BMI (measured BMI) to the one based on reported height and weight (measured height and weight). In the tables, height, weight and BMI are those measured by professional tailors at the survey sites. For both genders, reported BMI is slightly larger than measured BMI on average.

In addition to the bio-metric measurements, the data contains other variables for individual characteristics and socio-economic backgrounds. Education grouped into nine categories is 16.29 years for males and 15.75 years for females on average. Experience is calculated as potential experience = age − education − 6 and its mean is 17.54 years for males and 18.62 years for females. Fitness is defined as exercise hours per week. Its mean and median are 4.24 hours and 2.5 hours, respectively, for males. For females, its mean and median are 3.74 hours and 2.5 hours, respectively.

The data also include the number of children. Marital status is classified as three groups: single, married, divorced/widowed. Occupation consists of white collar, management, blue collar, and service. Race has four groups, including White, Hispanic, Black, and Asian. Birth region is grouped into five groups, including Midwest, Northeast, South, West, and Foreign. The majority in the dataset are white collar married white males and females born in the Midwest. As we will discuss later, the data also contains 40 body measures that include height and weight. The list of body measures are provided in Table S.3.

## Reporting Errors in Height and Weight

The following equation estimates the personal background that explains reporting error in height and weight:

$$\text{Reporting Error}_i^H = \pi X_i + \mu \text{Height}_i + \varepsilon_i, \tag{S.1}$$

$$\text{Reporting Error}_i^W = \pi X_i + \mu \text{Weight}_i + \varepsilon_i, \tag{S.2}$$

where $X_i$ is a set of covariates, including family income, age, age squared, occupation, education, marital status, fitness, race, and birth region. $\text{Height}_i$ is the true height in millimeters, and $\text{Weight}_i$ is the true weight in kilograms. We found dependence between reporting errors and some covariates. Table S.4 reports the estimation results. The standard errors are estimated by bootstrapping and are reported inside the parentheses. In equation (S.1), the coefficient of the true height is not statistically significant for both genders. We observe different results across genders. For males, family income is negatively correlated with the reporting error in height at the 1% significance level. Age squared is positively correlated with the reporting error. Hispanic males are more likely to under-report their height than White males. Males who were born in the Northeast are more likely to over-report their height than those from the Midwest. On the other hand, the coefficient of family income is not statistically significant for females. Older females are more likely to under-report their height. The estimation results are summarized in Fig S.1.

In equation (S.2), true weight is negatively correlated with the reporting error in weight (at the 1% significance level) for both genders: heavier people have a tendency to under-report their weight. For females, interestingly the coefficient of fitness is statistically significant at the 5% significance level and it is negatively correlated with the reporting-error in weight. Thus, females who spend more time on exercise have a tendency to under-report their weight. However, we find little evidence that other

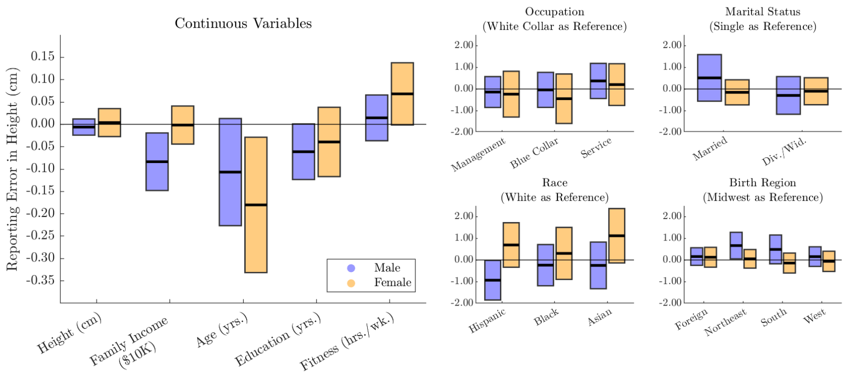


**Fig S.1. Personal background and reporting errors in height.** Estimated coefficients and bootstrapped 90% confidence bands are reported. Note that the unit for height is converted into centimeter (cm).

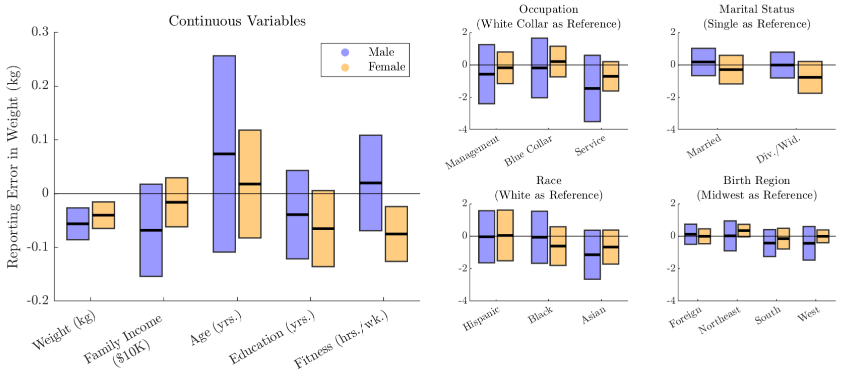


**Fig S.2. Personal background and reporting errors in weight.** Estimated coefficients and bootstrapped 90% confidence bands are reported.

personal background are correlated with reporting error in weight. The estimation results are summarized in Fig S.2.

Conditional quantile function is a useful tool to estimate heterogeneity in a conditional distribution. It also measures the proportion of reporting errors that are positive or negative. Fig S.3-S.4 present the estimation of the conditional quantiles of the reporting errors in height and weight conditional on the true measures, namely, $Q_\tau[\text{Reporting Error}^H \mid \text{Height}]$ and $Q_\tau[\text{Reporting Error}^W \mid \text{Weight}]$ for $\tau \in (0, 1)$, with their 95% confidence bands, respectively. We estimate the conditional quantiles using nonlinear polynomial regression. The figures display the median and the 10%, 25%, 75%, and 90% quantiles across genders. In Fig S.3, the results show that there is heterogeneity in the conditional distribution of the reporting error in height for both genders. Over-reporting of height is more pronounced for males than females. We notice that more than 75% of the sample of the males over-report their height. Interestingly, the median regression lines in both genders are approximately parallel with the horizontal line, which implies that the conditional median of the reporting error is independent of the true height. Bollinger also found that median regressions for earnings will be more robust to the reporting error than mean regressions [1]. Thus, it would be more natural to impose a restriction on the conditional quantile of the reporting error of

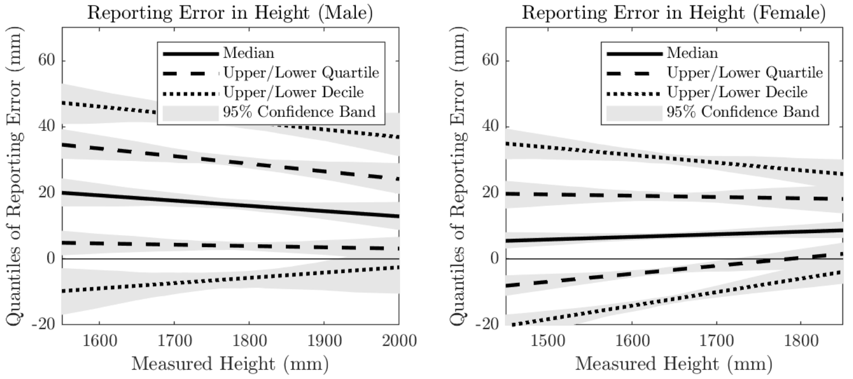


**Fig S.3. Conditional quantile of reporting errors in height conditional on true height.**

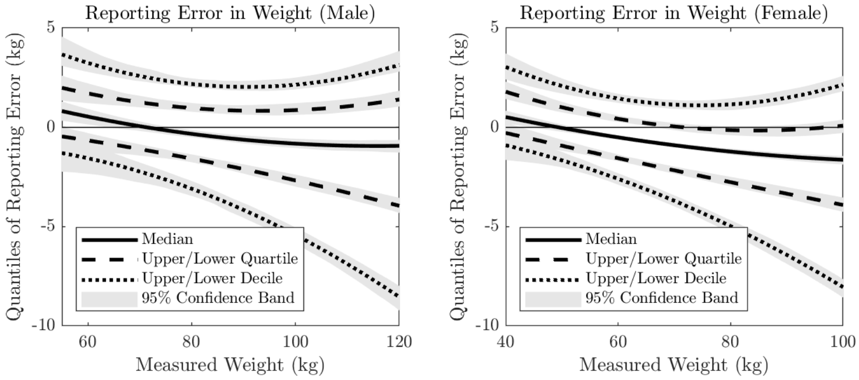


**Fig S.4. Conditional quantile of reporting errors in weight conditional on true weight.**

height than the conditional mean (e.g., Hu and Schennach [2] and Song [3]).

Fig S.4 also displays apparent heterogeneity in the conditional distribution for both genders. It shows that, for heavier than average people, the under-reporting of weight is more pronounced for females than males. We notice that within this group, almost 75% of the females under-report their weight. The median regressions in both genders are dependent on the level of the measured weight. This indicates that there are substantial nonclassical errors in the reported weight; therefore, a restriction on the conditional quantile may not be valid.

# Estimation of the Association between Physical Appearance and Labor Market Outcomes

## Height, Weight, and Reporting Errors

Most papers in the literature estimate the relationship between body shapes and family income by replacing body shapes with their observed proxies, such as height or weight. However, these measurements are hardly accurate to fully describe body shapes. Furthermore, including only height or weight without controlling for the other, as in the literature, could create an omitted variable problem. For instance, consider two people who have the same height but different weight. Comparing only height will not identify the difference in their body shapes. Thus, we consider the following two regression

equations:

$$\text{Family Income}_i = \alpha X_i + \beta_1 \text{Height}_i + \epsilon_i, \tag{S.3}$$
$$\text{Family Income}_i = \alpha X_i + \beta_1 \text{Height}_i + \beta_2 \text{Weight}_i + \epsilon_i, \tag{S.4}$$

where $X_i$ is a set of controls, including experience, squared experience, race, occupation, education, marital status, and number of children. We can test the importance of controlling for weight by comparing the estimated coefficients from the two equations. In addition, as mentioned, the data contains measurements on height and weight, both reported by subjects, and measured by on-site measurers. Therefore, by comparing estimates of measured ones with their reported counterparts, we can see the effect of reporting errors on the estimation results. Table S.5 reports the estimation results of reported height and weight. Table S.6 provides estimation results for measured height and weight.

The hypothesis that the coefficient on height is zero is tested across gender. The results for both genders are presented in each tables. Equation (S.3) of Table S.5 does not include reported weight. The column for males shows that education is statistically significant in the income equation. The coefficient of the reported height is positive and statistically significant at the 10% significance level. The column for females is somewhat different than that for males: the coefficient of experience, experience$^2$, and education are statistically significant. In addition, the coefficient on the reported height is positive and statistically significant at the 5% significance level. In equation (S.4), we add the reported weight to the set of regressors. The column for males shows that the coefficient of the reported height becomes statistically insignificant, and the coefficient of the reported weight is also insignificant. However, in the column for females, the coefficient of the reported height is still positive and statistically significant, but the coefficient on the reported weight is insignificant.

In Table S.6, we use the measured height and weight to estimate the income equation. Interestingly, the coefficients on the height for males in both equations are positive and statistically significant at the 1% significance level. Their magnitudes are larger than those from Table S.5. When the measured weight is added, its coefficient is still insignificant for males. For females, the coefficients on the measured height are statistically significant in both equations, and their magnitudes are larger than those from Table S.5. When the measured weight is added, its coefficient becomes significant at the 10% significance level, which shows a negative association between family income and weight. Thus, we confirm apparent reporting errors in height and weight. Particularly, the impacts of the reporting errors on the estimation results are more severe in males than females. These reporting errors introduce attenuation bias into the estimates. Furthermore, the estimation results from two equations (S.3-S.4) are different. It shows that using height only as a proxy to body shapes might be too simple to describe the delicate figures of the physical appearance. We refer to the main text for more results based on BMI.

To further investigate the role of the measurement errors on the body types, we run the following regression equation:

$$\text{Family Income}_i = \alpha X_i + \beta \text{Body}_i + \epsilon_i, \tag{S.5}$$

where $\text{Body}_i$ is a set of body measurements that include 40 measurements of various parts of the body. A full list of the measurements is provided in Table S.3. As these are more sophisticated than simple measurements of height and BMI, it is less likely that the measurement errors on body type are prevalent.

Fig S.5 presents the estimation results. Except height and weight, for brevity, we only report measures of body parts that are statistically significant. Interestingly, we

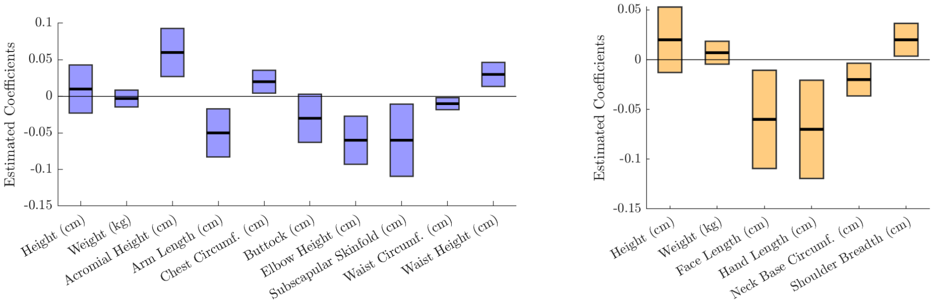


**Fig S.5. Various measures and family income.** Estimated coefficients and bootstrapped 90% confidence bands are reported for male (*left*) and female (*right*). Note that units for all measurements, except weight, are converted into centimeters (cm).

found eight statistically-significant body measurements for males and four for females. For instance, in the sample of males, Acromial Height (Sitting), Chest Circumference, and Waist Height (Preferred) have positive associations with the family income, while Arm Length (Shoulder-to-Elbow), Buttock (Knee Length), Elbow Height (Sitting), Subscapular Skinfold, Waist Circumference (Preferred) are negatively correlated with the family income. For females, Shoulder Breadth is positively correlated with the family income. However, the coefficients on Face Length, Hand Length, Neck Base Circumference are all negative. The most distinctive result is that the coefficients on height and weight for both genders are statistically insignificant in the regression. This implies that there are useful information on body types embedded into various body measures. The body shapes or types cannot be fully captured by simple measures such as height or weight.

Moreover, interactions between different body measurements are possible, as they have close relationships in constructing a body shape. Therefore, we consider the original regressors ($X_i$ and $\text{Body}_i$ in equation (S.5)) and interaction terms of $\text{Body}_i$ as a set of regressors. This gives 797 covariates, which makes the OLS regression inconsistent. We note that OLS is consistent under some regularity conditions only if the number of observations is larger than the number of covariates. To mitigate the issue of high-dimensional data, we use the following Lasso regression, which is valid under a sparsity assumption:

$$\min_{\psi} \left( \frac{1}{2N} \sum_{i=1}^{N} (\text{Family Income}_i - \psi Z_i)^2 + \lambda \sum_{j=1}^{p} |\psi_j| \right), \tag{S.6}$$

where $Z$ is a vector of covariates, including the interaction terms with size $p = 797$ and $\lambda \in [0, 1)$ is a regularization parameter. We construct the lasso fit using 10-fold cross-validation. Fig S.6 plots mean-squared-error (MSE) over the sequence of the regularization parameter $\lambda$ for each gender. For males, the minimum MSE is 0.231 at $\lambda = 0.024$ and the minimum MSE plus one standard error is 0.241 at $\lambda = 0.047$. For females, the minimum MSE is 0.216 at $\lambda = 0.017$ and the minimum MSE plus one standard error is 0.228 at $\lambda = 0.058$. The regression results show that many interaction terms are statistically significant. To save the space, we omit the results here. When equation (S.5) is re-estimated with these interaction terms (so-called "post-Lasso"), it obtains higher adjusted R squared (0.393 for males and 0.470 for females) than those in Fig S.5. Thus, it is highly likely that these body measures are interrelated. However, constructing stylized body types based on these relevant body measures is not straightforward and a nonstandard problem.

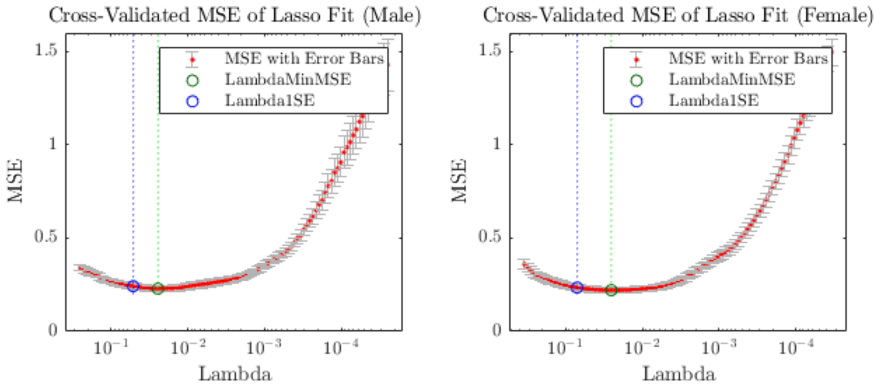


**Fig S.6. MSE and $\lambda$ in lasso.**

## Graphical Autoencoder

Mathematically, human body shapes can be represented as curved surfaces, or more formally manifolds $\mathcal{M}^{(i)}$ embedded in $\mathbb{R}^3$, where $i$ is an index identifying each individual. A manifold is a topological space that locally looks like Euclidean space near each point. The statistical models that this study considers are, in a generic form, the regression of an economic variable $Y$ with respect to a manifold-structured regressor $\mathcal{M}$ and other covariates $X$:

$$Y = \phi(\mathcal{M}, X; \theta) + \epsilon, \tag{S.7}$$

where $\phi$ is a known function up to unknown parameter $\theta$ and $\epsilon$ is an error term. Here, a problem rises regarding the manifold regressor $\mathcal{M}$, as the regressor $\mathcal{M}$ is an abstract, geometric object and not a usual vector variable, as in other typical economic and statistical models. In other words, there is no statistical model that naturally accepts the manifold regressor $\mathcal{M}$, unless $\mathcal{M}$ is somehow converted into a vector form.

Owing to the above bottleneck, one may consider measuring a few geometric dimensions, such as lengths and girths, and use those measurements to encode body shapes. However, as our study shows, such simplistic measurements are not an accurate characterization of complex geometric objects, such as human body shapes. Instead, data driven parameterizations such as in Wang, [7] Baek and Lee, [4] and Pishchulin *et al.* [8] provide more comprehensive and reliable codification of body shapes; however, many of these works assume that the human body shape distribution is linear, leading to inaccurate encoding of body shapes [5, 6].

In this study, we employ a data-driven, nonlinear parameterization of body shapes achieved via a graphical autoencoder. An autoencoder is a certain type of artificial neural network that possesses an hourglass shaped network architecture. An autoencoder can be thought of as two multilayer perceptron (MLP) models cascaded sequentially, where the first MLP codifies a high-dimensional input into a lower dimensional embedding (encoder) and the second MLP reconstructs the original input back from the encoded embedding (decoder). Because of the dimensional bottleneck created in the middle, the neural network is promoted to search for the most effective way of compressing the high dimensional input into the lower dimensional embedding.

Our proposed concept of a graphical autoencoder is an extension of such notion of autoencoders to manifold-structured data (see Fig 4 for a schematic overview). Similar to many geometric data analysis applications, we discretize a manifold $\mathcal{M}$ to a triangular mesh, achieving piece-wise linear approximation of the original surface. A triangular mesh is a graph $\mathcal{G} = \{\mathcal{V}, \mathcal{E}, \mathcal{F}\}$, where $\mathcal{V}$ is a set of vertices/nodes, $\mathcal{E}$ are edges interconnecting the vertices, and $\mathcal{F}$ are triangular facets. We equip the meshes $\mathcal{G}_{1,\cdots,N}$ with a semantic correspondence structure, such that the graph elements of the

same index correspond to the same body part location across all meshes $\mathcal{G}_{1,\cdots,N}$. This process is commonly called "registration" or "correspondence matching" in computer graphics and geometry processing literature and can be achieved via methods such as Zuffi and Black [9]; Wei *et al.* [10]; and Sun *et al.* [11,12].

In this setting, the graphical autoencoder is defined as follows:

$$\begin{aligned} p &= (f_1 \circ f_2 \circ \cdots \circ f_m)(V \in \mathcal{V}), \quad &\text{(encoder)} \\ V &= (g_1 \circ g_2 \circ \cdots \circ g_m)(p). \quad &\text{(decoder)} \end{aligned} \tag{S.8}$$

Here, each of the layers $f_1 \cdots f_m$ and $g_1 \cdots g_m$ are modeled as a simple perceptron:

$$f_i(h) \text{ or } g_i(h) = \sigma\left(\sum_j W_i^T h + b_i\right), \tag{S.9}$$

where $W_i$ are neural weights and $b_i$ are bias. $\sigma$ is the activation function, where we empirically decide to be rectified linear unit (ReLU) activation for $f_1 \cdots f_{m-1}$ and $g_1 \cdots g_{m-1}$. We set linear activation for the terminal layers $f_m$ and $g_m$ (*i.e.* no rectification).

Finally, we train the graphical autoencoder to minimize the mean square error between the original mesh and the reconstructed mesh:

$$\min_{\theta_f, \theta_g} \|V - g(p)\|, \tag{S.10}$$

where $p = f(V)$ by definition, $\theta_f$ and $\theta_g$ are the model parameters of $f$ and $g$ respectively, and $V$ is the list of vertex coordinates of a graphical model.

Note that the mean square error is not an ideal metric for shape dissimilarity. For example, it is widely known that the mean square error is not invariant to posture changes or other isometric transformations (e.g. rotations, parallel translations, etc.), implying that the two models with the exact same body shape but different postures or orientation may be deemed as different under the mean square error metric. However, instead of seeking an alternative metric, we employ the following provisional steps to maintain a reasonable computational load:

First, all scan data were normalized to have the same position, orientation, and posture. The centroid of each individual scan was set to (0,0,0). The anterior-posterior (AP) axis and the height (inferior-superior) axis were aligned with the z-axis and the y-axis, respectively. The body postures (joint angles) were adjusted to a standard "A" posture. The skeletal landmarks available in the CAESAR dataset were used for such joint angle alignment. Second, the geometric autoencoder was trained with randomly corrupted inputs, similar to the denoising autoencoders [13]. The random corruption (noise) added to the 3D scan data allows the network to develop more robust features. In a similar spirit, we also added dropout layers [14], with the dropout rate of 30%. Such stochastic perturbations help the autoencoder develop strong and robust shape descriptors. Lastly, we regularized the network weights with the $L_2$ regularizer to promote generalizable shape descriptors.

Empirically, the above provisions (i.e. data normalization, random corruption, and weight regularization) were effective in overcoming the limitations of the mean squared error. We tested the network through multiple training sessions with different initial network parameters. For each training session, we not only monitored the trend of the loss function but also inspected the graphical rendering of reconstructed 3D models. The training results were consistent both quantitatively and qualitatively from such observations.

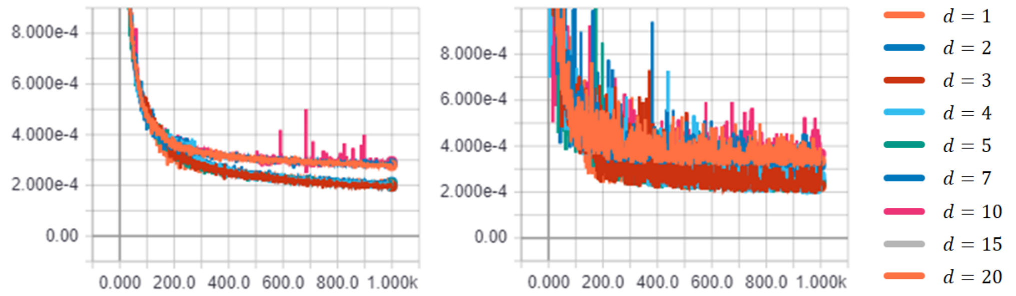


**Fig S.7. Result of training graphical autoencoder with the entire CAESAR dataset.** The abscissa is the number of epochs for the training and the ordinate is the model loss in terms of MSE. The left shows the loss on training dataset (training loss), while the right shows the loss on validation dataset (validation loss). The accuracy did not show any significant improvement after 1,000 epochs for all cases, and thus, we removed it from the figure for better visualization.

## Graphical Autoencoder on CAESAR Dataset

The first experiment was conducted to test the ability of the graphical autoencoder in embedding the geometric information underlying in the data. To achieve this, we applied the aforementioned graphical encoder to the entire CAESAR dataset, with varying embedding dimension $d$ from 1 to 20, as reported in Fig S.7. The embedding accuracy was below $3e^{-4}\text{m}^2$ in most cases. Particularly, when the dimension $d$ was 3, it showed the lowest MSE, in both training and validation losses, which justifies estimating $d = 3$ as the intrinsic dimension. As shown in Fig S.8, when the sample was divided into males and females, we found $d = 2$ for males and $d = 3$ for females.

For the meaning of the embedded parameters in the third dimension, the first component, $P_1$, discerned to be related to height of a person and $P_2$ to the body volume (obesity/leanness). Interestingly, as $P_3$ increases the body shape became more feminine, (namely, more prominent chest and hip-to-waist ratio) and, conversely, as it decreases the body shape became more masculine with less prominent chest and curves (see Fig S.9- S.13).

## Endogenous Obesity

One hurdle in estimating the relationship between physical appearance and income is the endogeneity concern associated with the body types, such as obesity and stature. To resolve the issue of endogenous obesity, we use the proxy variables approach, in which observed proxies to the unobserved common determinants of obesity and income control for the possible endogeneity in obesity. A set of the observed proxies includes fitness, car size, and birth state. Fitness is measured as hours of exercise per week. Car size is classified as two groups: Sedan (compact, economy, intermediate, full size, luxury, sports car) and Non-sedan (SUV, minivan, station wagon, truck, van). Birth state is classified as five groups: Foreign, West, Midwest, South, and Northeast. We choose these variables as relevant proxies to the unobserved common determinants of obesity, as fitness and car size could be related to personality and individual preference to body types, and birth state could reflect local and childhood nutrition environments that are correlated with individual obesity. To control for endogeneity in obesity, we assume the conditional independence of obesity and unobserved determinants of family income, conditional on the observed proxies, as commonly used in the literature on treatment effects.

We estimate equation (7) in the main text by controlling for the various subsets of

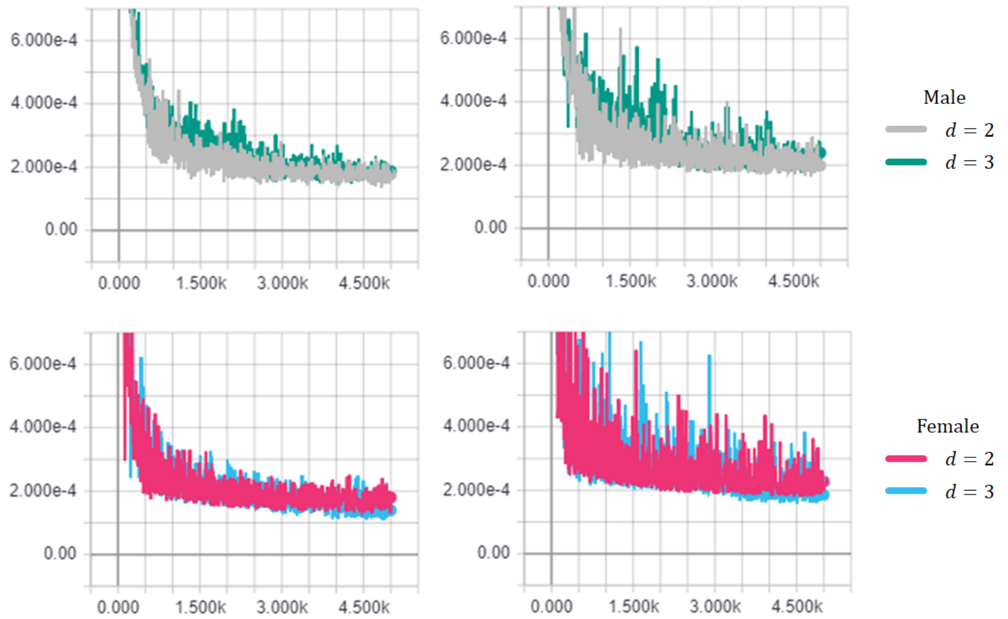


**Fig S.8. Result of training graphical autoencoder separately on each gender.** The abscissa is the number of epochs for the training and the ordinate is the model loss in terms of MSE. The left shows the loss on training dataset (training loss) while the right shows the loss on validation dataset (validation loss).

the proxy variables. For comparison, we use measured Height, BMI, and hip-to-waist Ratio in place of $P_1$, $P_2$, and $P_3$, respectively. The estimation results are reported in Table S.11. The estimated coefficients of height are similar to those in Table S.8 for both genders (i.e., columns for equation (4) in the main text). In particular, the coefficient of height is still positive in the equation for both genders. On the other hand, the estimated coefficient of BMI for females becomes negative and significant in the female subsample.

Table S.12 reports the estimation results for the main text equation (7) with $P_1$, $P_2$, and $P_3$ instead. Notably, fitness and car size are not statistically significant; however, birth state (Northeast) is statistically significant. When all proxy variables are included, the estimated coefficient of $P_1$ is 0.054 for males. It is statistically significant at the 1% significance level. Thus, taller males tend to have higher family income. However, we do not find a statistically meaningful relationship between male obesity and the family income. For females, the $P_2$ measurement is negatively associated with the family income, and its coefficient is statistically significant at the 1% significance level. Thus, we find that female obesity matters for the family income in a negative sense; however their stature and hip-to-waist ratio are not associated with family income. The results are qualitatively similar to those from Table S.10, where obesity is assumed to be exogenous.

## Endogenous Stature

Here, we conjecture that $P_1$ or stature would possibly be the endogenous regressor. To address this issue, we look for a set of valid instrumental variables (IV). Our identification strategy is to assume that unobserved determinants of Shoe size, Pants size, and Jacket Size for males (or Blouse Size for females) are uncorrelated with individual cognitive and noncognitive abilities. Given the condition, we adopt these

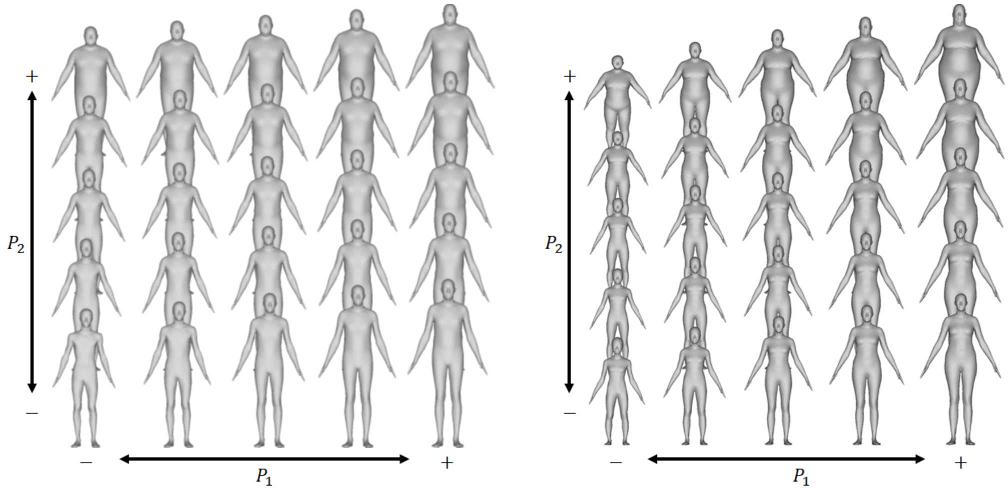


**Fig S.9. Body shape parameters derived from the graphical autoencoder.** 3D body shape models for male (left) and female (right) are arranged in accordance with their body shape parameters, with increments of -3$\sigma$, -1.5$\sigma$, 0, 1.5$\sigma$, and 3$\sigma$ with respect to the mean in each direction, where $\sigma$ is the s.d. of each parameter.

unobserved size determinants or variations as valid IVs and incorporate the instrumental variables or control functions (CF) approach into the economic model based on the graph convolution method.

Fig S.14 shows a graphical depiction of the causal diagram (see *e.g.*, Pearl [15] and Chalak and White [16]). Complete circles denote observed variables and dashed circles denote unobserved determinants. Arrows denote direct causal relations. A line without arrows denotes dependence between two variables. The primary parameter of interest is the association between body type and family income. However, body type (stature) is endogenous because of the dependence of body type determinants and cognitive and noncognitive abilities. Shoe size, jacket (blouse) size, and pants size are determined by body type as well as unobserved determinants, such as personal size preference. We assume that such unobserved size determinants are uncorrelated with ability and that they are excluded from family income. Given these conditions, they can serve as legitimate IVs to identify the causal relation. Indeed, it is plausible to assume that these variables are legitimate IVs. First, they are unlikely to be correlated with the unobserved determinants of family income, such as cognitive and noncognitive abilities. For instance, it is natural to assume that highly capable people do not necessarily wear bigger shoes or pants given their foot length or waist circumference. Furthermore, it is unlikely that these IVs directly cause the family income. Second, as shown in the estimation results below, they are also strongly correlated with the body type.

As each size determinants or variations are unobserved, we estimate them from the projection of the observed size on the most relevant body part. In practice, we consider the projection of the reported shoe size, pants size and jacket (or blouse size) on the measured foot length, waist circumference, and chest circumference, respectively. The residuals from each projection are used as the estimated size determinants or variations (analogous to the 'residuals as instruments' in Hausman and Taylor [17]).

We consider the following first-step reduced form equation:

$$\begin{aligned} P_{1i} = \delta X_i &+ \gamma_1 \text{Shoe Size Determinants}_i \\ &+ \gamma_2 \text{Jacket Size/Blouse Size Determinants}_i \\ &+ \gamma_3 \text{Pants Size Determinants}_i + \nu_i, \end{aligned} \tag{S.11}$$

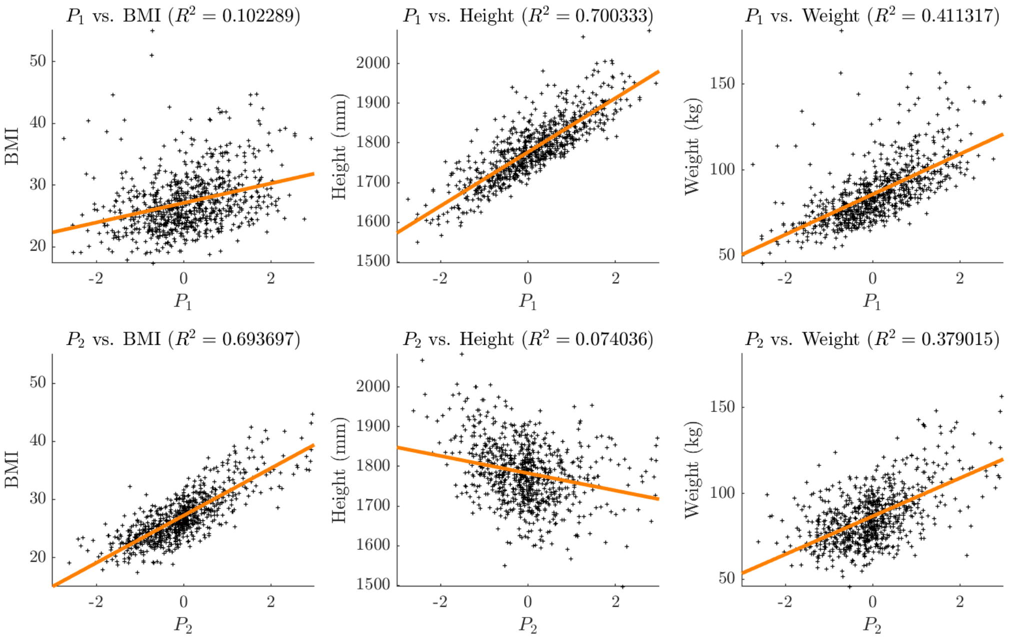


**Fig S.10. Relationship between body shape parameters and the classical body measurements for males.** The straight line displays the linear fit. The R-squared is reported in the parentheses.

where Shoe Size Determinants$_i$ is the estimated individual $i$'s variation or determinants in shoe size and Jacket Size/Blouse Size Determinants$_i$ is the estimated variation in jacket size for males (or blouse size for females), and Pants Size Determinants$_i$ is variation in pants size, and where $X_i$ are a set of exogenous regressors and $\nu_i$ is idiosyncratic shocks to the $P_{1i}$. By construction, $\nu_i$ is the component that generates the endogeneity. From the reduced-from equations (S.11), we estimate the control function $\hat{\nu}_i$. In the second-step, we then estimate the income equation by adding the control function as follows:

$$\begin{cases} \text{Family Income}_i = \alpha X_i + \beta_1 P_{1i} + \beta_2 P_{2i} + \pi\hat{\nu}_i + \epsilon_i & \text{if male,} \\ \text{Family Income}_i = \alpha X_i + \beta_1 P_{1i} + \beta_2 P_{2i} + \beta_3 P_{3i} + \pi\hat{\nu}_i + \epsilon_i & \text{if female.} \end{cases} \quad \text{(S.12)}$$

As $\hat{\nu}$ corrects for the sources of the endogeneity, we can consistently estimate the parameters associated with the physical appearances. Another advantage of the control functions approach is that we can test whether the physical appearances are endogenous by checking if $\pi = 0$.

Table S.14 reports estimation results for the equations (S.11–S.12). In the columns for the equation (S.11), all IVs are statistically significant and positively correlated with stature in both genders. Experience is also positively associated with individual's stature and the relation is nonlinear. People who were born in foreign countries or the Northeast are likely to be shorter than those born in the Midwest. For males, education is negatively correlated with stature. Asian people are less likely to be taller than White people. For females, Hispanic and Asian people are less likely to be taller than White people.

In the columns for the equation (S.12), the estimated coefficient of $P_1$ is 0.097 for males, which is larger than that in Table S.12. It is statistically significant at the 5% significance level. Thus, taller males tend to have higher family income. Interestingly, we do not find a statistically significant relationship between male obesity and family

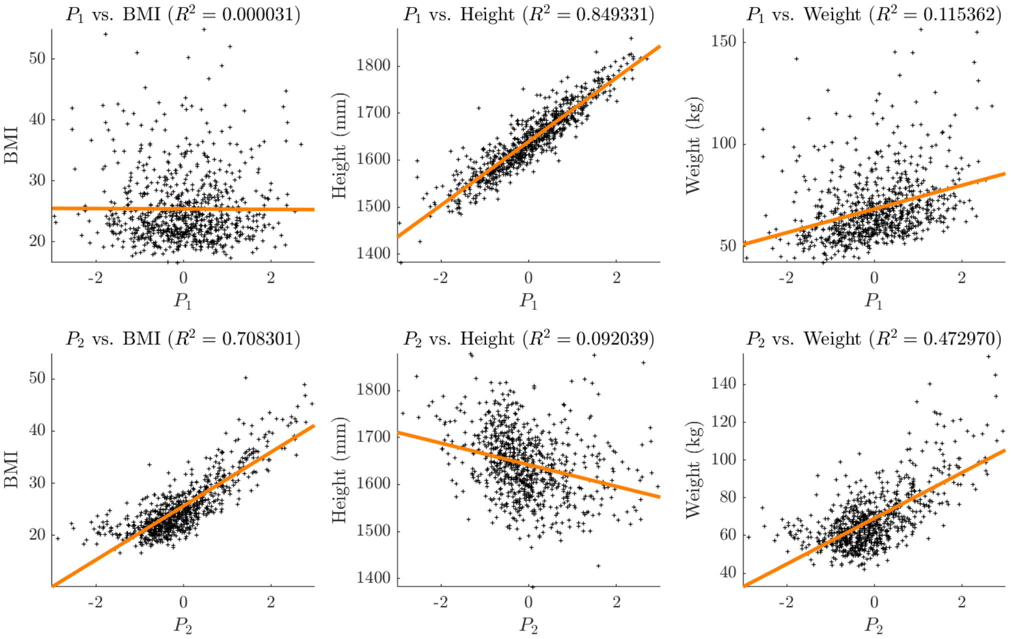


**Fig S.11. Relationship between body shape parameters and the classical body measurements for females.** The straight line displays the linear fit. The R-squared is reported in the parentheses.

income. We estimate that one standard deviation increase in $P_1$ measurement is associated with $\$0.097 \times 70,000 = \$6,790$ increase in the family income for a male who earns $\$70,000$ of median family income. This is equivalent to $\$\frac{0.097}{6.8} \times 70,000 = \$998.5$ increase in family income per centimeter. Note that for males, one standard deviation in $P_1$ is equivalent to 6.8 centimeters in height and one standard deviation in $P_2$ is equivalent to $4.07kg/m^2$ in BMI.

The estimation results for the covariates resemble those in previous tables. As shown in the literature on the returns to education, education has a positive impact on family income. Its estimated coefficient is 0.046 and it is statistically significant at the 1% significance level. Males born in the Northeast tend to have higher family income than those born in the Midwest. Interestingly, the estimated coefficient of $\hat{\nu}_1$ is statistically insignificant. Thus, we find no strong evidence that $P_1$ is a endogenous regressor.

For females, the estimated coefficient of $P_1$ is negative and the estimated coefficient of $P_3$ is positive, but they are not statistically significant. The $P_2$ measurement is negatively associated with family income. Its coefficient is $-0.069$, and it is statistically significant at the 1% significance level. Thus, we find that female obesity is negatively correlated with her family income, but her stature or hip-to-waist ratio is not. One standard deviation decrease in $P_2$ measurement is associated with $\$0.069 \times 70,000 = \$4,830$ increase in family income for a female who earns $\$70,000$ family income. This can be interpreted as $\$\frac{0.069}{5.17} \times 70,000 = \$934.2$ increase per one unit of BMI. Note that for females one standard deviation in $P_1$ is equivalent to 6.8 centimeters in height and one standard deviation in $P_2$ is equivalent to $5.17kg/m^2$ in BMI.

For females, experience is positively correlated with family income. As commonly reported in the literature on the wage equation, the experience displays a quadratic functional form. Education has a positive impact on family income and its coefficient is statistically significant at the 1% significance level, which is similar to the finding for males. Similarly, females born in the Northeast tend to have higher family income than

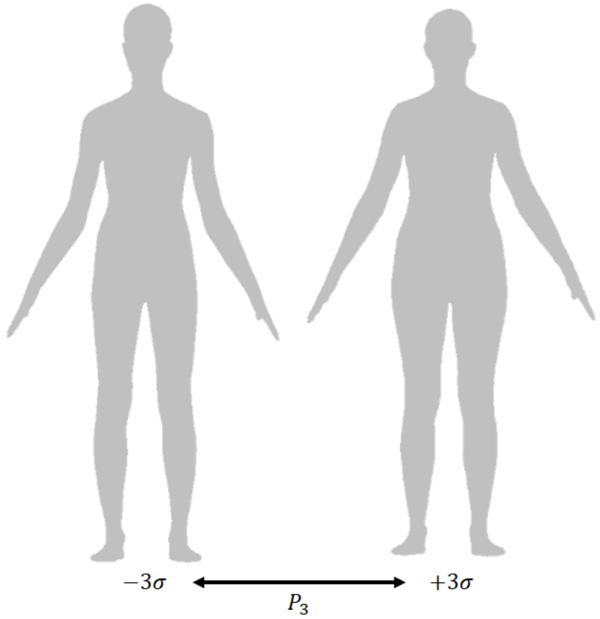


**Fig S.12. The third body shape parameter $P_3$ for females.** The third parameter tends to capture the hip-to-waist ratio of the body shape among the female subsample.

those born in the Midwest. The estimated coefficient of $\hat{\nu}$ is statistically significant. Thus, we find substantial evidence that stature is endogenous in the female's income equation.

For comparison, we apply the control functions approach to income equations, where height, BMI, and hip-to-waist ratio are used in place of the extracted body features. The corresponding reduced-form and structural equations are the same as the equations (S.11–S.12). Table S.13 reports the estimation results. For males, we estimate that one centimeter increase in height is associated with $\$0.01 \times 70,000 = \$700$ increase in family income for a male who earns $\$70,000$ of median family income. The estimated relationship is smaller than that when the extracted features from the deep learning are used. For females, pants size is not associated with height in the first step regression. The estimated coefficient of $\hat{\nu}$ is significant in the female sample, which implies evidence of endogenous female height. For females, interestingly, coefficients of height, BMI, and hip-to-waist ratio are all insignificant. Thus, we do not find strong evidence of the association between body shape and family income when using height, BMI, and hip-to-waist ratio. Consequently, we observe that the estimation results with the classical measurements are volatile across different regression models—OLS, proxy variable approach, and control functions approach. However, those with the deep-learned body parameters are very stable across different models, and interestingly, capture gender differentials in the impact of body types on income.

# References

1. Bollinger CR. Measurement Error in the Current Population Survey: A Nonparametric Look. Journal of Labor Economics. 1998;16:576–594.

2. Hu Y, Schennach SM. Instrumental Variable Treatment of Nonclassical Measurement Error Models. Econometrica. 2008;76:195–216.

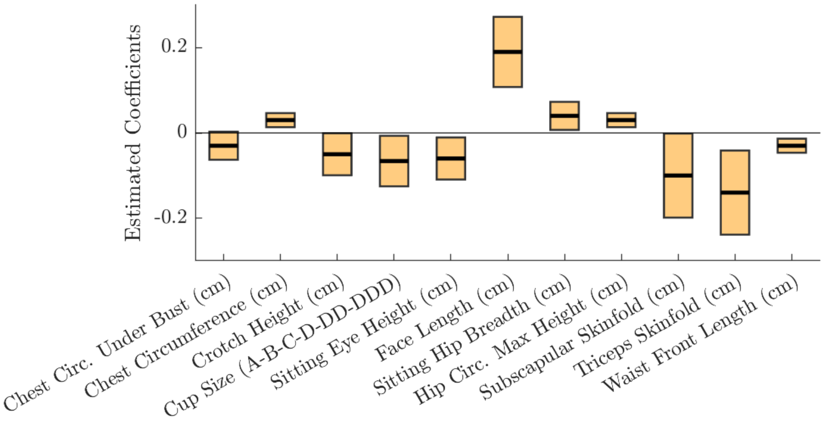


**Fig S.13. Various measures and $P_3$ for females.** Estimated coefficients and bootstrapped 90% confidence bands are reported for females. Note that units for all measurements, except cup size, are converted into centimeters (cm).


3. Song S. Semiparametric Estimation of Models with Conditional Moment Restrictions in the Presence of Nonclassical Measurement Errors. Journal of Econometrics. 2015;185:95–109.

4. Baek S, Lee K. Parametric human body shape modeling framework for human-centered product design. Computer-Aided Design. 2012;44:56–67.

5. Baek SY. Nonlinear Analysis of the Space of Human Body Shapes and Its Application to Parametric Human Modeling System. PhD Dissertation, Seoul National University. 2013.

6. Freifeld O, Black MJ. Lie Bodies: A Manifold Representation of 3D Human Shape. In: European Conference on Computer Vision (ECCV). Part I, LNCS 7572. Springer-Verlag; 2012. p. 1–14.

7. Wang CCL. Parameterization and parametric design of mannequins. Computer-Aided Design. 2005;37(1):83–98.

8. Pishchulin L, Wuhrer S, Helten T, Theobalt C, Schiele B. Building statistical shape spaces for 3D human modeling. Pattern Recognition. 2017;67:276–286.

9. Zuffi S, Black MJ. The stitched puppet: A graphical model of 3D human shape and pose. In: Proceedings of the IEEE Conference on Computer Vision and Pattern Recognition; 2015. p. 3537–3546.

10. Wei L, Huang Q, Ceylan D, Vouga E, Li H. Dense human body correspondences using convolutional networks. In: Proceedings of the IEEE Conference on Computer Vision and Pattern Recognition; 2016. p. 1544–1553.

11. Sun Z, He Y, Gritsenko A, Lendasse A, Baek S. Embedded spectral descriptors: learning the point-wise correspondence metric via Siamese neural networks. Journal of Computational Design and Engineering. 2020;7(1):18–29.

12. Sun Z, Rooke E, Charton J, He Y, Lu J, Baek S. ZerNet: Convolutional Neural Networks on Arbitrary Surfaces Via Zernike Local Tangent Space Estimation. Computer Graphics Forum. 2020;39(6):204-216.

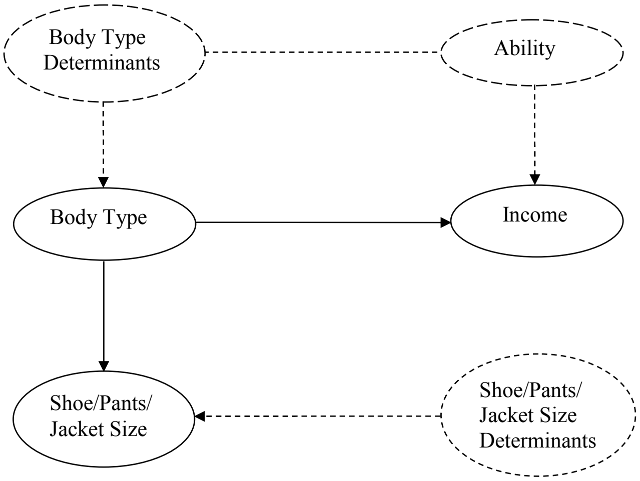


**Fig S.14. Causal diagram for the impact of body type on income.** Shoe/Pants/Jacket size determinants are independent of individual ability; therefore, that they can serve as valid instrumental variables.

13. Vincent P, Larochelle H, Bengio Y, Manzagol PA. Extracting and composing robust features with denoising autoencoders. In: Proceedings of the 25th international conference on Machine learning; 2008. p. 1096–1103.

14. Srivastava N, Hinton G, Krizhevsky A, Sutskever I, Salakhutdinov R. Dropout: a simple way to prevent neural networks from overfitting. The journal of machine learning research. 2014;15(1):1929–1958.

15. Pearl J. Causality: Models, Reasoning, and Inference. New York, NY: Cambridge University Press; 2000.

16. Chalak K, White H. An extended class of instrumental variables for the estimation of causal effects. Canadian Journal of Economics. 2011;44:1–51.

17. Hausman JA, Taylor WE. Identification in Linear Simultaneous Equations Models with Covariance Restrictions: An Instrumental Variables Interpretation. Econometrica. 1983;51:1527–1550.

| Variable | Mean | Median | S.D. | Min | Max |
|---|---|---|---|---|---|
| Family Income ($) | 76,085 | 70,000 | 41,470 | 7,500 | 150,000 |
| Reported Height (mm) | 1,798.2 | 1,803.4 | 82.5 | 1,498.6 | 2,108.2 |
| Reported Weight (kg) | 86.0 | 83.9 | 17.3 | 48.5 | 188.2 |
| Reported BMI (kg/m$^2$) | 26.5 | 25.8 | 4.6 | 14.0 | 59.5 |
| Height (mm) | 1,782.6 | 1,778.5 | 78.1 | 1,497.0 | 2,084.0 |
| Weight (kg) | 86.8 | 83.9 | 17.5 | 45.8 | 181.4 |
| BMI (kg/m$^2$) | 27.2 | 26.4 | 4.8 | 17.4 | 55.1 |
| Experience (years) | 17.5 | 17.0 | 10.2 | 0 | 47.0 |
| Education (years) | 16.3 | 16.0 | 2.5 | 12.0 | 24.0 |
| # of Children | 1.3 | 1.0 | 1.4 | 0 | 7.0 |
| Fitness (hours) | 4.2 | 2.5 | 3.0 | 0.5 | 10.0 |

| Variable | # of Samples | Variable | # of Samples |
|---|---|---|---|
| Marital Status (Single) | 240 | Race (White) | 644 |
| Marital Status (Married) | 473 | Race (Hispanic) | 18 |
| Marital Status (Div./Wid.) | 61 | Race (Black) | 68 |
| Birth Region (Foreign) | 159 | Race (Asian) | 44 |
| Birth Region (Midwest) | 275 | Occupation (White Collar) | 461 |
| Birth Region (Northeast) | 106 | Occupation (Management) | 144 |
| Birth Region (South) | 106 | Occupation (Blue Collar) | 101 |
| Birth Region (West) | 128 | Occupation (Service) | 6 |
| # of Total Observations | 774 | | |

**Table S.1. Summary statistics (male).**

| Variable | Mean | Median | S.D. | Min | Max |
|---|---|---|---|---|---|
| Family Income ($) | 65,998 | 52,500 | 38,853 | 7,500 | 150,000 |
| Reported Height (mm) | 1,649.6 | 1651.0 | 76.1 | 1,320.8 | 1,930.4 |
| Reported Weight (kg) | 67.9 | 63.5 | 16.9 | 37.2 | 172.3 |
| Reported BMI (kg/m$^2$) | 24.9 | 23.3 | 5.9 | 12.9 | 57.8 |
| Height (mm) | 1,642.2 | 1,640.0 | 71.3 | 1,382.0 | 1,879.0 |
| Weight (kg) | 68.8 | 64.9 | 17.3 | 39.2 | 156.5 |
| BMI (kg/m$^2$) | 25.5 | 23.8 | 6.1 | 15.2 | 57.1 |
| Experience (years) | 18.6 | 19.0 | 10.8 | 0 | 50.0 |
| Education (years) | 15.8 | 16.0 | 2.1 | 12.0 | 24.0 |
| # of Children | 1.0 | 0 | 1.2 | 0 | 6.0 |
| Fitness (hours) | 3.7 | 2.5 | 2.7 | 0.5 | 10.0 |

| Variable | # of Samples | Variable | # of Samples |
|---|---|---|---|
| Marital Status (Single) | 248 | Race (White) | 644 |
| Marital Status (Married) | 407 | Race (Hispanic) | 11 |
| Marital Status (Div./Wid.) | 134 | Race (Black) | 88 |
| Birth Region (Foreign) | 105 | Race (Asian) | 46 |
| Birth Region (Midwest) | 318 | Occupation (White Collar) | 607 |
| Birth Region (Northeast) | 103 | Occupation (Management) | 52 |
| Birth Region (South) | 122 | Occupation (Blue Collar) | 49 |
| Birth Region (West) | 141 | Occupation (Service) | 81 |
| **# of Total Observations** | 789 | | |

**Table S.2. Summary statistics (female).**

| Variables (mm) | |
|---|---|
| Acromial Height, Sitting | Head Length |
| Ankle Circumference | Hip Breadth, Sitting |
| Arm Length (Spine to Wrist) | Hip Circumference, Maximum |
| Arm Length (Shoulder to Wrist) | Hip Circumference Max Height |
| Arm Length (Shoulder to Elbow) | Knee Height |
| Armscye Circumference (Scye Circumference Over Acromion) | Neck Base Circumference |
| Bizygomatic Breadth | Shoulder Breadth |
| Chest Circumference | Sitting Height |
| Bust/Chest Circumference Under Bust | Height |
| Buttock-Knee Length | Subscapular Skinfold |
| Chest Girth at Scye (Chest Circumference at Scye) | Thigh Circumference |
| Crotch Height | Thigh Circumference Max Sitting |
| Elbow Height, Sitting | Thumb Tip Reach |
| Eye Height, Sitting | Triceps Skinfold |
| Face Length | Total Crotch Length (Crotch Length) |
| Foot Length | Vertical Trunk Circumference |
| Hand Circumference | Waist Circumference, Preferred |
| Hand Length | Waist Front Length |
| Head Breadth | Waist Height, Preferred |
| Head Circumference | Weight (kg) |

**Table S.3. List of various body measures.**

| Variable | Error in Height (Eq. (S.1)) Male | Error in Height (Eq. (S.1)) female | Error in Weight (Eq. (S.2)) Male | Error in Weight (Eq. (S.2)) female |
|---|---|---|---|---|
| Intercept | 114.481*** | 41.826 | 8.574** | 4.056* |
| | (36.056) | (38.067) | (3.438) | (2.329) |
| Height (mm) | -0.006 | 0.004 | | |
| | (0.011) | (0.019) | | |
| Weight (kg) | | | -0.056*** | -0.040*** |
| | | | (0.018) | (0.015) |
| Family Income | -5.847*** | -0.105 | -0.477 | -0.112 |
| | (2.738) | (1.810) | (0.365) | (0.194) |
| Age | -1.067 | -1.801* | 0.074 | 0.018 |
| | (0.728) | (0.920) | (0.111) | (0.061) |
| Age$^2$ | 0.014* | 0.021** | -7.6e-4 | -1.0e-6 |
| | (0.008) | (0.011) | (0.001) | (7.1e-4) |
| Occupation (Management) | -1.401 | -2.376 | -0.566 | -0.172 |
| | (4.335) | (6.439) | (1.105) | (0.592) |
| Occupation (Blue Collar) | -0.398 | -4.486 | -0.181 | 0.211 |
| | (4.924) | (6.960) | (1.115) | (0.573) |
| Occupation (Service) | 3.736 | 2.051 | -1.444 | -0.698 |
| | (4.938) | (5.863) | (1.241) | (0.549) |
| Education | -0.612 | -0.393 | -0.039 | -0.065 |
| | (0.378) | (0.471) | (0.050) | (0.043) |
| Marital Status (Married) | 5.146 | -1.538 | 0.185 | -0.285 |
| | (6.544) | (3.527) | (0.511) | (0.535) |
| Marital Status (Div./Wid.) | -2.960 | -1.026 | -0.003 | -0.761 |
| | (5.293) | (3.790) | (0.483) | (0.596) |
| Fitness | 0.146 | 0.682 | 0.020 | -0.075** |
| | (0.311) | (0.423) | (0.054) | (0.031) |
| Race (Hispanic) | -9.340* | 6.936 | -0.036 | 0.045 |
| | (5.558) | (6.252) | (0.975) | (0.948) |
| Race (Black) | -2.383 | 3.050 | -0.066 | -0.610 |
| | (5.788) | (7.302) | (0.974) | (0.723) |
| Race (Asian) | -2.506 | 11.208 | -1.143 | -0.670 |
| | (6.554) | (7.640) | (0.916) | (0.636) |
| Birth Region (Foreign) | 1.597 | 1.277 | 0.119 | -0.008 |
| | (2.469) | (2.792) | (0.376) | (0.279) |
| Birth Region (Northeast) | 6.668* | 0.546 | 0.019 | 0.344 |
| | (3.728) | (2.612) | (0.559) | (0.234) |
| Birth Region (South) | 4.893 | -1.405 | -0.425 | -0.151 |
| | (4.043) | (2.807) | (0.504) | (0.388) |
| Birth Region (West) | 1.562 | -0.617 | -0.438 | -0.009 |
| | (2.754) | (2.841) | (0.628) | (0.239) |
| $\bar{R}^2$ | 0.011 | 0.009 | 0.034 | 0.070 |
| $F$-statistic vs. constant model | 1.47 | 1.42 | 2.50 | 4.33 |
| $p$-value | 0.094 | 0.114 | 0.001 | 6.4e-09 |
| $N$ | 778 | 793 | 776 | 792 |

**Table S.4. The association between reporting error in height/weight and personal background.**

| Variable | Income (Eq. (S.3)) Male | Income (Eq. (S.3)) Female | Income (Eq. (S.4)) Male | Income (Eq. (S.4)) Female |
|---|---|---|---|---|
| Intercept | 9.108*** (0.517) | 8.747*** (0.510) | 9.259*** (0.529) | 8.666*** (0.521) |
| Reported Height (mm) | 4.0e-4* (2.2e-4) | 5.3e-4** (2.1e-4) | 2.3e-4 (2.7e-4) | 6.5e-4*** (2.2e-4) |
| Reported Weight (kg) | | | 0.002 (0.001) | -0.002 (0.001) |
| Covariates | ✓ | ✓ | ✓ | ✓ |
| $\bar{R}^2$ | 0.410 | 0.407 | 0.334 | 0.410 |
| $F$-statistic vs. constant model | 31.3 | 43.8 | 29.2 | 40.7 |
| $p$-value | 1.9e-62 | 6.1e-84 | 5.4e-62 | 3.7e-83 |
| $N$ | 790 | 801 | 788 | 799 |

**Table S.5. The association between reported height/weight and family income.**

| Variable | Income (Eq. (S.3)) Male | Income (Eq. (S.3)) Female | Income (Eq. (S.4)) Male | Income (Eq. (S.4)) Female |
|---|---|---|---|---|
| Intercept | 8.608*** (0.509) | 8.573*** (0.535) | 8.775*** (0.505) | 8.466*** (0.539) |
| Height (mm) | 6.8e-4*** (2.2e-4) | 6.4e-4*** (2.4e-4) | 5.0e-4** (2.5e-4) | 7.9e-4*** (2.5e-4) |
| Weight (kg) | | | 0.002 (0.001) | -0.002* (0.001) |
| Covariates | ✓ | ✓ | ✓ | ✓ |
| $\bar{R}^2$ | 0.338 | 0.412 | 0.339 | 0.414 |
| $F$-statistic vs. constant model | 32.0 | 44.2 | 29.9 | 41.4 |
| $p$-value | 1.0e-63 | 1.0e-84 | 2.1e-63 | 1.4e-84 |
| $N$ | 791 | 802 | 791 | 802 |

**Table S.6. The association between height/weight and family income.**

| Variable | Income (Main Text Eq. (2)) Male | Income (Main Text Eq. (2)) Female | Income (Main Text Eq. (3)) Male | Income (Main Text Eq. (3)) Female | Income (Main Text Eq. (4)) Male | Income (Main Text Eq. (4)) Female |
|---|---|---|---|---|---|---|
| Intercept | 9.685*** (0.332) | 9.747*** (0.393) | 9.668*** (0.336) | 9.746*** (0.389) | 8.965*** (0.523) | 8.885*** (0.544) |
| Reported BMI | 0.005 (0.004) | -0.005 (0.003) | -0.007 (0.009) | -0.019*** (0.007) | 0.005 (0.005) | -0.004 (0.003) |
| Reported Height (mm) | | | | | 3.9e-4* (2.1e-4) | 5.2e-4** (2.3e-4) |
| Reported Weight (kg) | | | 0.004* (0.002) | 0.005** (0.002) | | |
| Covariates | ✓ | ✓ | ✓ | ✓ | ✓ | ✓ |
| $\bar{R}^2$ | 0.332 | 0.407 | 0.334 | 0.410 | 0.334 | 0.410 |
| $F$-statistic vs. constant model | 31.1 | 43.1 | 29.2 | 40.6 | 29.2 | 40.7 |
| $p$-value | 4.5e-62 | 7.7e-83 | 5.7e-62 | 4.9e-83 | 5.4e-62 | 3.7e-83 |
| $N$ | 788 | 799 | 788 | 799 | 788 | 799 |

**Table S.7. The association between reported BMI and family income.**

| Variable | Income (Main Text Eq. (2)) Male | Income (Main Text Eq. (2)) Female | Income (Main Text Eq. (3)) Male | Income (Main Text Eq. (3)) Female | Income (Main Text Eq. (4)) Male | Income (Main Text Eq. (4)) Female |
|---|---|---|---|---|---|---|
| Intercept | 9.678*** (0.345) | 9.751*** (0.382) | 9.678*** (0.342) | 9.768*** (0.384) | 8.499*** (0.511) | 8.737*** (0.537) |
| BMI | 0.006 (0.004) | -0.005* (0.003) | -0.018** (0.009) | -0.024*** (0.007) | 0.005 (0.004) | -0.005* (0.003) |
| Height (mm) | | | | | 6.6e-4*** (2.2e-4) | 6.3e-4*** (2.4e-4) |
| Weight (kg) | | | 0.007*** (0.002) | 0.007** (0.003) | | |
| Covariates | ✓ | ✓ | ✓ | ✓ | ✓ | ✓ |
| $\bar{R}^2$ | 0.332 | 0.410 | 0.339 | 0.414 | 0.338 | 0.414 |
| $F$-statistic vs. constant model | 31.2 | 43.8 | 29.9 | 41.4 | 29.9 | 41.4 |
| $p$-value | 2.1e-62 | 5.2e-84 | 1.7e-63 | 1.5e-84 | 2.3e-63 | 1.4e-84 |
| $N$ | 791 | 802 | 791 | 802 | 791 | 802 |

**Table S.8. The association between BMI and family income.**

| Variable | Income (Main Text Eq. (5)) | | Income (Main Text Eq. (6)) | | Income (Main Text Eq. (7)) | |
|---|---|---|---|---|---|---|
| | Male | Female | Male | Female | Male | Female |
| Intercept | 8.608***<br>(0.492) | 8.573***<br>(0.534) | 9.680***<br>(0.347) | 9.751***<br>(0.386) | 8.499***<br>(0.513) | 8.490***<br>(0.625) |
| Height<br>(mm) | 6.8e-4***<br>(2.1e-4) | 6.4e-4***<br>(2.4e-4) | | | 6.6e-4***<br>(2.2e-4) | 6.2e-4**<br>(2.4e-4) |
| BMI | | | 0.006<br>(0.004) | -0.005*<br>(0.003) | 0.005<br>(0.004) | -0.004<br>(0.003) |
| Hip-to-waist<br>Ratio | | | | | | 0.002<br>(0.002) |
| Covariates | ✓ | ✓ | ✓ | ✓ | ✓ | ✓ |
| $\bar{R}^2$ | 0.338 | 0.412 | 0.332 | 0.410 | 0.338 | 0.413 |
| $F$-statistic vs. constant model | 32.0 | 44.2 | 31.2 | 43.8 | 29.9 | 38.5 |
| $p$-value | 1.0e-63 | 1.0e-84 | 2.1e-62 | 5.2e-84 | 2.3e-63 | 2.1e-83 |
| $N$ | 791 | 802 | 791 | 802 | 791 | 799 |

**Table S.9. The association between BMI/height/hip-to-waist-ratio and family income.**

| Variable | Income (Main Text Eq. (5)) | | Income (Main Text Eq. (6)) | | Income (Main Text Eq. (7)) | |
|---|---|---|---|---|---|---|
| | Male | Female | Male | Female | Male | Female |
| Intercept | 9.823***<br>(0.309) | 9.629***<br>(0.392) | 9.841***<br>(0.307) | 9.620***<br>(0.392) | 9.823***<br>(0.317) | 9.638***<br>(0.382) |
| $P_1$ | 0.052***<br>(0.020) | 0.033*<br>(0.018) | | | 0.052***<br>(0.019) | 0.024<br>(0.020) |
| $P_2$ | | | 2.0e-4<br>(0.002) | -0.056***<br>(0.017) | -0.002<br>(0.019) | -0.052***<br>(0.018) |
| $P_3$ | | | | | | 0.014<br>(0.020) |
| Covariates | ✓ | ✓ | ✓ | ✓ | ✓ | ✓ |
| $\bar{R}^2$ | 0.337 | 0.410 | 0.330 | 0.415 | 0.336 | 0.415 |
| $F$-statistic vs. constant model | 31.9 | 43.8 | 31.0 | 44.7 | 29.5 | 38.9 |
| $p$-value | 1.6e-63 | 5.4e-84 | 6.9e-62 | 1.9e-85 | 9.4e-63 | 3.0e-84 |
| $N$ | 791 | 802 | 791 | 802 | 791 | 802 |

**Table S.10. The association between body-type parameters and family income.**

| Variable | Income (Main Text Eq. (7)) Male | Female | Income (Main Text Eq. (7)) Male | Female | Income (Main Text Eq. (7)) Male | Female |
|---|---|---|---|---|---|---|
| Intercept | 8.482***<br>(0.511) | 8.626***<br>(0.585) | 8.510***<br>(0.513) | 8.804***<br>(0.571) | 8.521***<br>(0.501) | 8.711***<br>(0.596) |
| Height (mm) | 6.5e-4***<br>(2.1e-4) | 5.8e-4**<br>(2.4e-4) | 7.2e-4***<br>(2.0e-4) | 5.2e-4**<br>(2.3e-4) | 6.9e-4***<br>(2.2e-4) | 5.6e-4**<br>(2.4e-4) |
| BMI | 0.006<br>(0.004) | -0.005*<br>(0.003) | 0.005<br>(0.004) | -0.007**<br>(0.003) | 0.005<br>(0.004) | -0.007**<br>(0.003) |
| Hip-to-waist Ratio | | 0.001<br>(0.002) | | 0.001<br>(0.002) | | 0.002<br>(0.002) |
| Covariates | ✓ | ✓ | ✓ | ✓ | ✓ | ✓ |
| Fitness | 0.005<br>(0.006) | -0.004<br>(0.006) | 0.004<br>(0.006) | -0.001<br>(0.006) | 0.006<br>(0.006) | -5.9e-4<br>(0.006) |
| Car Size (Sedan) | | | -0.012<br>(0.033) | -0.014<br>(0.037) | -0.006<br>(0.033) | -0.016<br>(0.037) |
| Birth State (Foreign) | | | | | 0.022<br>(0.043) | -0.025<br>(0.059) |
| Birth State (Northeast) | | | | | 0.109*<br>(0.057) | 0.125**<br>(0.051) |
| Birth State (South) | | | | | 0.009<br>(0.061) | 0.047<br>(0.046) |
| Birth State (west) | | | | | 0.029<br>(0.052) | -0.011<br>(0.051) |
| $\bar{R}^2$ | 0.336 | 0.414 | 0.335 | 0.413 | 0.335 | 0.413 |
| $F$-statistic vs. constant model | 27.5 | 35.9 | 24.8 | 32.3 | 19.9 | 26.3 |
| $p$-value | 7.2e-62 | 3.6e-82 | 9.9e-59 | 1.7e-77 | 3.4e-56 | 4.5e-75 |
| $N$ | 786 | 792 | 759 | 757 | 750 | 754 |

**Table S.11. The association between BMI/height/hip-to-waist-ratio and family income - Proxy variable approach.**

| Variable | Income (Main Text Eq. (7)) | | Income (Main Text Eq. (7)) | | Income (Main Text Eq. (7)) | |
|---|---|---|---|---|---|---|
| | Male | Female | Male | Female | Male | Female |
| Intercept | 9.819*** (0.315) | 9.638*** (0.403) | 9.937*** (0.303) | 9.669*** (0.367) | 9.912*** (0.303) | 9.680*** (0.378) |
| $P_1$ | 0.054*** (0.019) | 0.020 (0.020) | 0.054*** (0.019) | 0.016 (0.019) | 0.054*** (0.019) | 0.019 (0.019) |
| $P_2$ | 0.002 (0.018) | -0.059*** (0.017) | -0.007 (0.019) | -0.070*** (0.018) | -0.004 (0.018) | -0.071*** (0.018) |
| $P_3$ | | 0.005 (0.020) | | 0.004 (0.020) | | 0.009 (0.019) |
| Covariates | ✓ | ✓ | ✓ | ✓ | ✓ | ✓ |
| Fitness | 0.004 (0.006) | -0.004 (0.006) | 0.004 (0.006) | -0.002 (0.006) | 0.006 (0.006) | -0.001 (0.006) |
| Car Size (Sedan) | | | -0.017 (0.060) | -0.014 (0.036) | -0.011 (0.033) | -0.016 (0.037) |
| Birth State (Foreign) | | | | | 0.021 (0.046) | 0.021 (0.057) |
| Birth State (Northeast) | | | | | 0.111** (0.054) | 0.123** (0.051) |
| Birth State (South) | | | | | 0.014 (0.059) | 0.055 (0.048) |
| Birth State (west) | | | | | 0.032 (0.051) | -0.010 (0.050) |
| $\bar{R}^2$ | 0.334 | 0.416 | 0.332 | 0.418 | 0.333 | 0.418 |
| $F$-statistic vs. constant model | 27.2 | 36.4 | 24.5 | 33.0 | 19.7 | 26.9 |
| $p$-value | 2.5e-61 | 3.3e-83 | 5.3e-58 | 4.3e-79 | 1.5e-55 | 1.1e-76 |
| $N$ | 786 | 795 | 759 | 760 | 750 | 757 |

**Table S.12. The association between body-type parameters and family income - Proxy variable approach.**

| Variable | Height (1st-step Eq. (S.11)) | | Income (2nd-step Eq. (S.12)) | |
|---|---|---|---|---|
| | Male | Female | Male | Female |
| Intercept | 1832.318*** (32.212) | 1634.629*** (27.136) | 7.785*** (1.104) | 10.674*** (1.294) |
| Height (mm) | | | 0.001* (5.9e-4) | -6.4e-4 (7.5e-4) |
| BMI | | | 0.004 (0.004) | -0.005 (0.003) |
| Hip-to-waist Ratio | | | | 0.002 (0.002) |
| $\hat{\nu}$ | | | -6.0e-4 (6.5e-4) | 0.001* (8.0e-4) |
| Shoe Size | 13.502*** (4.958) | 19.847*** (4.187) | | |
| Jacket Size (Blouse Size) | 10.132*** (1.309) | 7.425*** (1.874) | | |
| Pants Size | 8.372*** (2.384) | 1.010 (1.416) | | |
| Covariates | ✓ | ✓ | ✓ | ✓ |
| Proxy Variables | ✓ | ✓ | ✓ | ✓ |
| $\bar{R}^2$ | 0.228 | 0.203 | 0.327 | 0.404 |
| $F$-statistic vs. constant model | 10.7 | 10.1 | 17.0 | 24.1 |
| $p$-value | 3.39e-29 | 9.4e-28 | 5.8e-47 | 1.1e-68 |
| $N$ | 660 | 716 | 660 | 716 |

**Table S.13. The association between BMI/height/hip-to-waist-ratio and family income - Control function approach.**

| Variable | $P_1$ (1st-step Eq. (S.11)) | | Income (2nd-step Eq. (S.12)) | |
|---|---|---|---|---|
| | Male | Female | Male | Female |
| Intercept | 0.546<br>(0.410) | -0.181<br>(0.347) | 9.945***<br>(0.336) | 9.735***<br>(0.365) |
| $P_1$ | | | 0.097**<br>(0.049) | -0.074<br>(0.056) |
| $P_2$ | | | 6.5e-4<br>(0.019) | -0.069***<br>(0.018) |
| $P_3$ | | | | 0.009<br>(0.020) |
| $\hat{\nu}$ | | | -0.058<br>(0.055) | 0.101*<br>(0.058) |
| Shoe Size | 0.159**<br>(0.062) | 0.193***<br>(0.053) | | |
| Jacket Size<br>(Blouse Size) | 0.118***<br>(0.016) | 0.099**<br>(0.025) | | |
| Pants Size | 0.123***<br>(0.030) | 0.040**<br>(0.020) | | |
| Covariates | ✓ | ✓ | ✓ | ✓ |
| Proxy Variables | ✓ | ✓ | ✓ | ✓ |
| $\bar{R}^2$ | 0.217 | 0.203 | 0.325 | 0.408 |
| $F$-statistic vs. constant model | 10.1 | 10.2 | 16.9 | 24.6 |
| $p$-value | 1.9e-27 | 8.1e-28 | 1.4e-46 | 6.9e-70 |
| $N$ | 660 | 718 | 660 | 718 |

**Table S.14. The association between body-type parameters and family income - Control function approach.**
