# Supplementary figures and images for: Body shape matters: Evidence from machine learning on body shape-income relationship
Source: PLoS One. 2021 Jul 30;16(7):e0254785. doi: 10.1371/journal.pone.0254785 (PMC8323889; doi:10.1371/journal.pone.0254785)

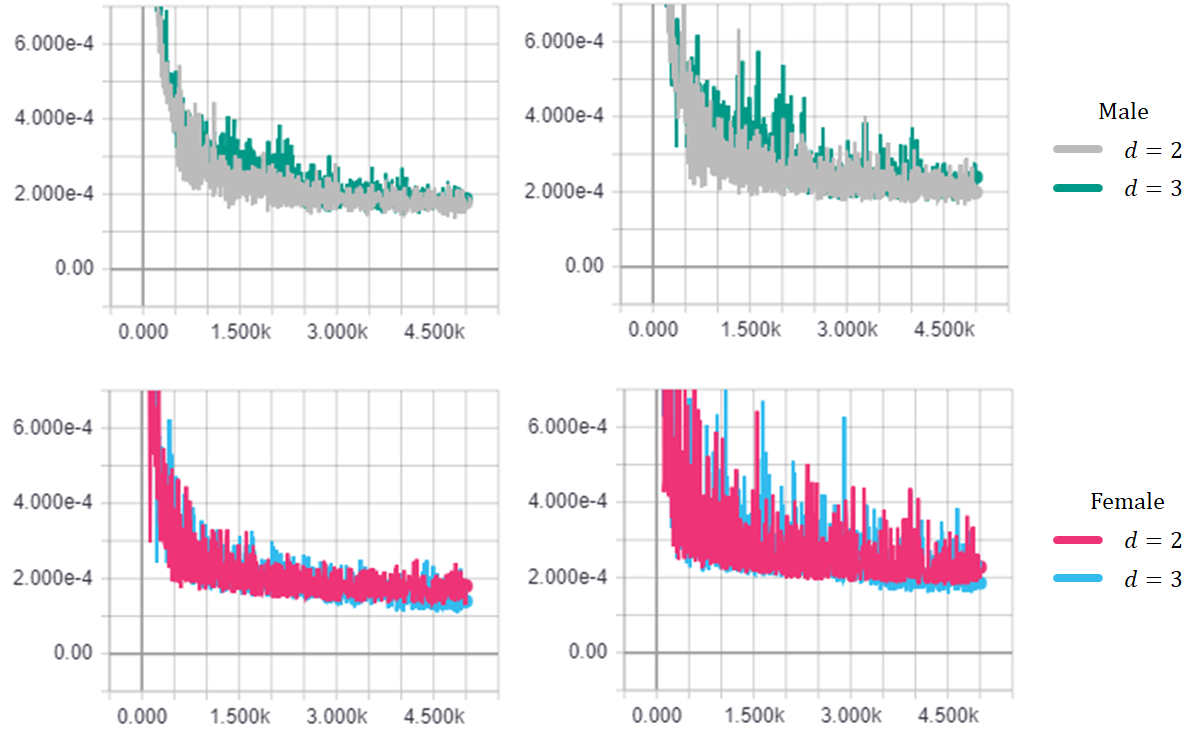

Supplement: S1 Fig — The abscissa is the number of epochs for the training and the ordinate is the model loss in terms of MSE. The left shows the loss on training dataset (training loss) while the right shows the loss on validation dataset (validation loss). (TIF) [file pone.0254785.s003.tif]

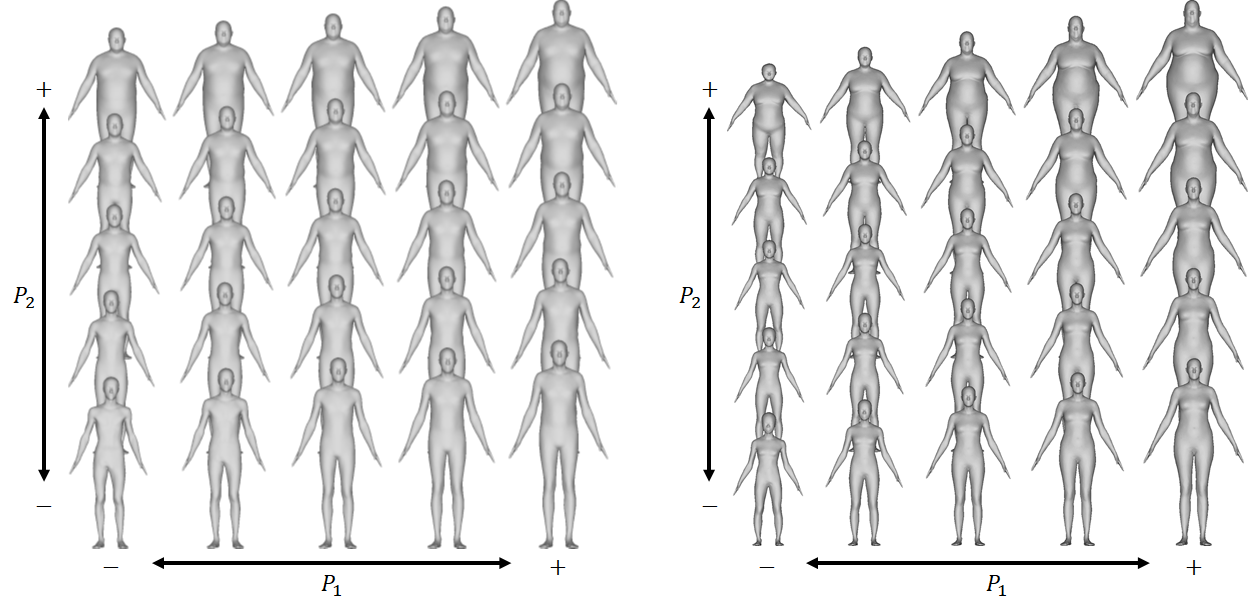

Supplement: S2 Fig — 3D body shape models for male (left) and female (right) are arranged in accordance with their body shape parameters, with increments of -3σ, -1.5σ, 0, 1.5σ, and 3σ with respect to the mean in each direction, where σ is the s.d. of each parameter. (TIF) [file pone.0254785.s004.tif]

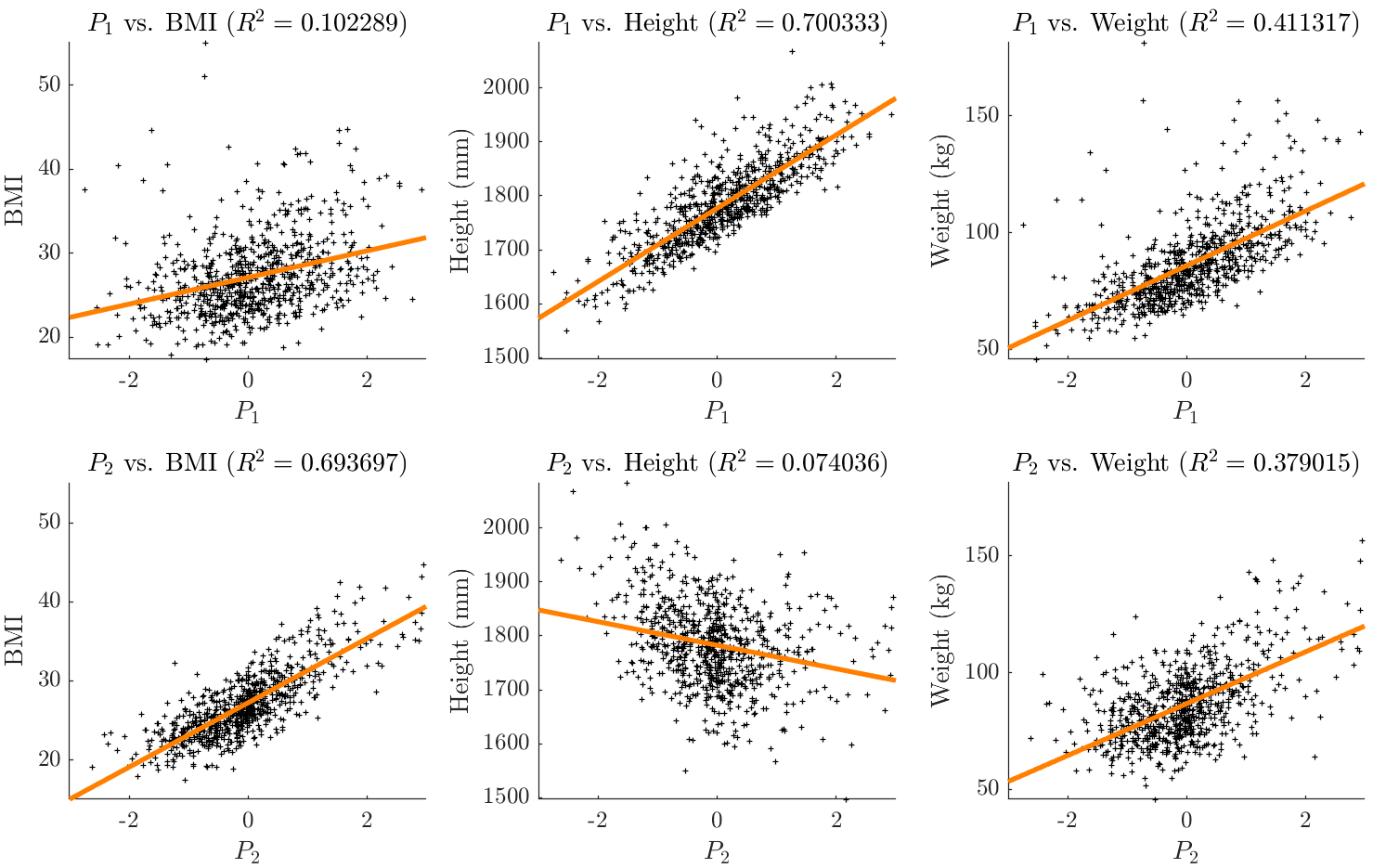

Supplement: S3 Fig — The straight line displays the linear fit. The R-squared is reported in the parentheses. (TIF) [file pone.0254785.s005.tif]

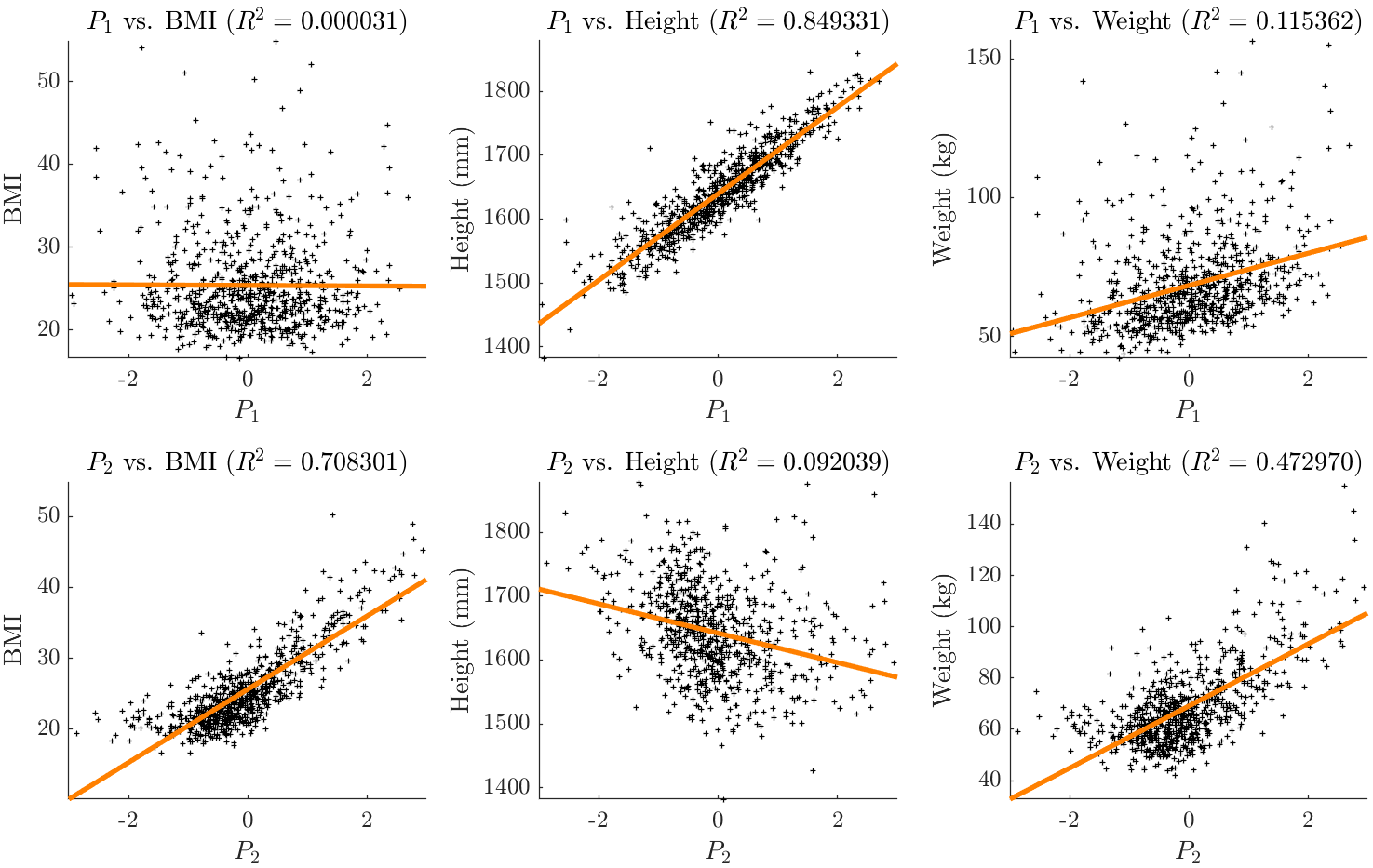

Supplement: S4 Fig — The straight line displays the linear fit. The R-squared is reported in the parentheses. (TIF) [file pone.0254785.s006.tif]

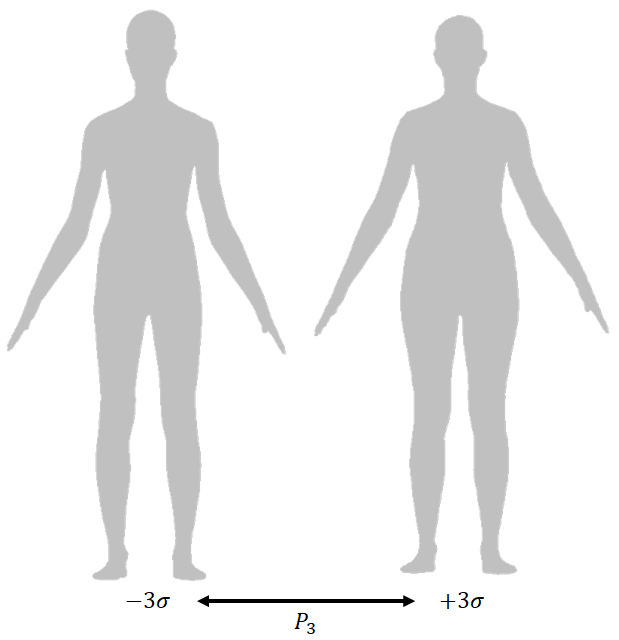

Supplement: S5 Fig — The third parameter tends to capture the hip-to-waist ratio of the body shape among the female subsample. (TIF) [file pone.0254785.s007.tif]

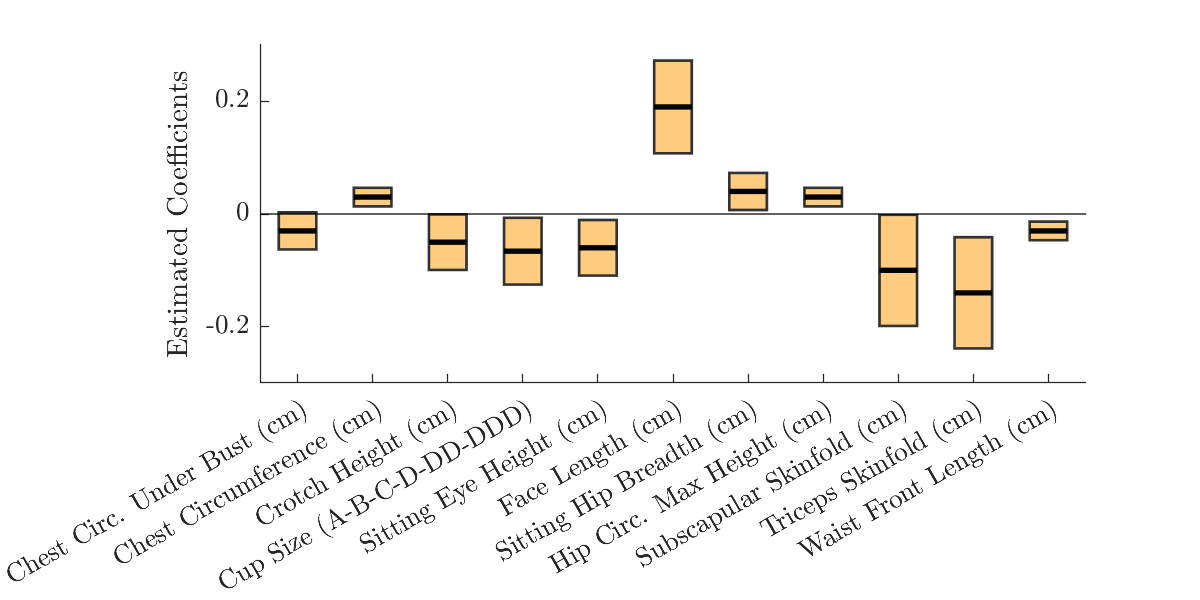

Supplement: S6 Fig — Estimated coefficients and bootstrapped 90% confidence bands are reported for females. Note that units for all measurements, except cup size, are converted into centimeters (cm). (TIF) [file pone.0254785.s008.tif]
