# Supplementary material for: Body shape matters: Evidence from machine learning on body shape-income relationship
Source: PLoS One. 2021 Jul 30;16(7):e0254785. doi: 10.1371/journal.pone.0254785 (PMC8323889; doi:10.1371/journal.pone.0254785)
Supplement: S1 Table — (PDF) [file pone.0254785.s009.pdf]

Variable	Income (Eq. (2)) Male	Income (Eq. (2)) Female	Income (Eq. (3)) Male	Income (Eq. (3)) Female	Income (Eq. (4)) Male	Income (Eq. (4)) Female
Intercept	9.685***<br>(0.332)	9.747***<br>(0.393)	9.668***<br>(0.336)	9.746***<br>(0.389)	8.965***<br>(0.523)	8.885***<br>(0.544)
Reported BMI	0.005<br>(0.004)	-0.005<br>(0.003)	-0.007<br>(0.009)	-0.019***<br>(0.007)	0.005<br>(0.005)	-0.004<br>(0.003)
Reported Height (mm)					3.9e-4*<br>(2.1e-4)	5.2e-4**<br>(2.3e-4)
Reported Weight (kg)			0.004*<br>(0.002)	0.005**<br>(0.002)		
Covariates	✓	✓	✓	✓	✓	✓
$\bar{R}^2$	0.332	0.407	0.334	0.410	0.334	0.410
F-statistic vs. constant model	31.1	43.1	29.2	40.6	29.2	40.7
p-value	4.5e-62	7.7e-83	5.7e-62	4.9e-83	5.4e-62	3.7e-83
N	788	799	788	799	788	799

S1 Table. The association between reported BMI and family income.
