# Supplementary material for: Body shape matters: Evidence from machine learning on body shape-income relationship
Source: PLoS One. 2021 Jul 30;16(7):e0254785. doi: 10.1371/journal.pone.0254785 (PMC8323889; doi:10.1371/journal.pone.0254785)
Supplement: S2 Table — (PDF) [file pone.0254785.s010.pdf]

Variable	Income (Eq. (2))		Income (Eq. (3))		Income (Eq. (4))	
	Male	Female	Male	Female	Male	Female
Intercept	9.678*** (0.345)	9.751*** (0.382)	9.678*** (0.342)	9.768*** (0.384)	8.499*** (0.511)	8.737*** (0.537)
BMI	0.006 (0.004)	-0.005* (0.003)	-0.018** (0.009)	-0.024*** (0.007)	0.005 (0.004)	-0.005* (0.003)
Height (mm)					6.6e-4*** (2.2e-4)	6.3e-4*** (2.4e-4)
Weight (kg)			0.007*** (0.002)	0.007** (0.003)		
Covariates	✓	✓	✓	✓	✓	✓
$\bar{R}^2$	0.332	0.410	0.339	0.414	0.338	0.414
F-statistic vs. constant model	31.2	43.8	29.9	41.4	29.9	41.4
p-value	2.1e-62	5.2e-84	1.7e-63	1.5e-84	2.3e-63	1.4e-84
N	791	802	791	802	791	802

S2 Table. The association between BMI and family income.
