# Supplementary material for: Body shape matters: Evidence from machine learning on body shape-income relationship
Source: PLoS One. 2021 Jul 30;16(7):e0254785. doi: 10.1371/journal.pone.0254785 (PMC8323889; doi:10.1371/journal.pone.0254785)
Supplement: S3 Table — (PDF) [file pone.0254785.s011.pdf]

Variables (mm)	
Acromial Height, Sitting	Head Length
Ankle Circumference	Hip Breadth, Sitting
Arm Length (Spine to Wrist)	Hip Circumference, Maximum
Arm Length (Shoulder to Wrist)	Hip Circumference Max Height
Arm Length (Shoulder to Elbow)	Knee Height
Armscye Circumference (Scye Circumference Over Acromion)	Neck Base Circumference
Bizygomatic Breadth	Shoulder Breadth
Chest Circumference	Sitting Height
Bust/Chest Circumference Under Bust	Height
Buttock-Knee Length	Subscapular Skinfold
Chest Girth at Scye (Chest Circumference at Scye)	Thigh Circumference
Crotch Height	Thigh Circumference Max Sitting
Elbow Height, Sitting	Thumb Tip Reach
Eye Height, Sitting	Triceps Skinfold
Face Length	Total Crotch Length (Crotch Length)
Foot Length	Vertical Trunk Circumference
Hand Circumference	Waist Circumference, Preferred
Hand Length	Waist Front Length
Head Breadth	Waist Height, Preferred
Head Circumference	Weight (kg)

S3 Table. List of various body measures.
