# Supplementary material for: Body shape matters: Evidence from machine learning on body shape-income relationship
Source: PLoS One. 2021 Jul 30;16(7):e0254785. doi: 10.1371/journal.pone.0254785 (PMC8323889; doi:10.1371/journal.pone.0254785)
Supplement: S4 Table — (PDF) [file pone.0254785.s012.pdf]

Variable	Income (Eq. (5)) Male	Income (Eq. (5)) Female	Income (Eq. (6)) Male	Income (Eq. (6)) Female	Income (Eq. (7)) Male	Income (Eq. (7)) Female
Intercept	9.823*** (0.309)	9.629*** (0.392)	9.841*** (0.307)	9.620*** (0.392)	9.823*** (0.317)	9.638*** (0.382)
P_1	0.052*** (0.020)	0.033* (0.018)			0.052*** (0.019)	0.024 (0.020)
P_2			2.0e-4 (0.002)	-0.056*** (0.017)	-0.002 (0.019)	-0.052*** (0.018)
P_3						0.014 (0.020)
Covariates	✓	✓	✓	✓	✓	✓
$\bar{R}^2$	0.337	0.410	0.330	0.415	0.336	0.415
F-statistic vs. constant model	31.9	43.8	31.0	44.7	29.5	38.9
p-value	1.6e-63	5.4e-84	6.9e-62	1.9e-85	9.4e-63	3.0e-84
N	791	802	791	802	791	802

S4 Table. The association between body-type parameters and family income.
