# Supplementary material for: Body shape matters: Evidence from machine learning on body shape-income relationship
Source: PLoS One. 2021 Jul 30;16(7):e0254785. doi: 10.1371/journal.pone.0254785 (PMC8323889; doi:10.1371/journal.pone.0254785)
Supplement: S5 Table — (PDF) [file pone.0254785.s013.pdf]

Variable	Income (Eq. (5)) Male	Income (Eq. (5)) Female	Income (Eq. (6)) Male	Income (Eq. (6)) Female	Income (Eq. (7)) Male	Income (Eq. (7)) Female
Intercept	8.608***<br>(0.492)	8.573***<br>(0.534)	9.680***<br>(0.347)	9.751***<br>(0.386)	8.499***<br>(0.513)	8.490***<br>(0.625)
Height (mm)	6.8e-4***<br>(2.1e-4)	6.4e-4***<br>(2.4e-4)			6.6e-4***<br>(2.2e-4)	6.2e-4**<br>(2.4e-4)
BMI			0.006<br>(0.004)	-0.005*<br>(0.003)	0.005<br>(0.004)	-0.004<br>(0.003)
Hip-to-waist Ratio						0.002<br>(0.002)
Covariates	✓	✓	✓	✓	✓	✓
$\bar{R}^2$	0.338	0.412	0.332	0.410	0.338	0.413
F-statistic vs. constant model	32.0	44.2	31.2	43.8	29.9	38.5
p-value	1.0e-63	1.0e-84	2.1e-62	5.2e-84	2.3e-63	2.1e-83
N	791	802	791	802	791	799

S5 Table. The association between BMI/height/hip-to-waist-ratio and family income.
