# Supplementary material for: Body shape matters: Evidence from machine learning on body shape-income relationship
Source: PLoS One. 2021 Jul 30;16(7):e0254785. doi: 10.1371/journal.pone.0254785 (PMC8323889; doi:10.1371/journal.pone.0254785)
Supplement: S6 Table — (PDF) [file pone.0254785.s014.pdf]

Variable	Height (1st-step Eq. (S.11)) Male	Height (1st-step Eq. (S.11)) Female	Income (2nd-step Eq. (S.12)) Male	Income (2nd-step Eq. (S.12)) Female
Intercept	1832.318***<br>(32.212)	1634.629***<br>(27.136)	7.785***<br>(1.104)	10.674***<br>(1.294)
Height (mm)			0.001*<br>(5.9e-4)	-6.4e-4<br>(7.5e-4)
BMI			0.004<br>(0.004)	-0.005<br>(0.003)
Hip-to-waist Ratio				0.002<br>(0.002)
$\hat{\nu}$			-6.0e-4<br>(6.5e-4)	0.001*<br>(8.0e-4)
Shoe Size	13.502***<br>(4.958)	19.847***<br>(4.187)		
Jacket Size (Blouse Size)	10.132***<br>(1.309)	7.425***<br>(1.874)		
Pants Size	8.372***<br>(2.384)	1.010<br>(1.416)		
Covariates	✓	✓	✓	✓
Proxy Variables	✓	✓	✓	✓
$\bar{R}^2$	0.228	0.203	0.327	0.404
F-statistic vs. constant model	10.7	10.1	17.0	24.1
p-value	3.39e-29	9.4e-28	5.8e-47	1.1e-68
N	660	716	660	716

S6 Table. The association between BMI/height/hip-to-waist-ratio and family income - Control function approach.
