# Supplementary material for: Body shape matters: Evidence from machine learning on body shape-income relationship
Source: PLoS One. 2021 Jul 30;16(7):e0254785. doi: 10.1371/journal.pone.0254785 (PMC8323889; doi:10.1371/journal.pone.0254785)
Supplement: S7 Table — (PDF) [file pone.0254785.s015.pdf]

Variable	P_1 (1st-step Eq. (S.11))		Income (2nd-step Eq. (S.12))	
	Male	Female	Male	Female
Intercept	0.546<br>(0.410)	-0.181<br>(0.347)	9.945***<br>(0.336)	9.735***<br>(0.365)
P_1			0.097**<br>(0.049)	-0.074<br>(0.056)
P_2			6.5e-4<br>(0.019)	-0.069***<br>(0.018)
P_3				0.009<br>(0.020)
$\hat{\nu}$			-0.058<br>(0.055)	0.101*<br>(0.058)
Shoe Size	0.159**<br>(0.062)	0.193***<br>(0.053)		
Jacket Size<br>(Blouse Size)	0.118***<br>(0.016)	0.099**<br>(0.025)		
Pants Size	0.123***<br>(0.030)	0.040**<br>(0.020)		
Covariates	✓	✓	✓	✓
Proxy Variables	✓	✓	✓	✓
$\bar{R}^2$	0.217	0.203	0.325	0.408
F-statistic vs. constant model	10.1	10.2	16.9	24.6
p-value	1.9e-27	8.1e-28	1.4e-46	6.9e-70
N	660	718	660	718

S7 Table. The association between body-type parameters and family income - Control function approach.
